# Supplementary material for: Muscle calcium stress cleaves junctophilin1, unleashing a gene regulatory program predicted to correct glucose dysregulation
Source: eLife. 2023 Feb 1;12:e78874. doi: 10.7554/eLife.78874 (PMC9891728; doi:10.7554/eLife.78874)

**Figure 1-source data 1:** JPh1 raw blot shown in Fig 1A

MHN MHS


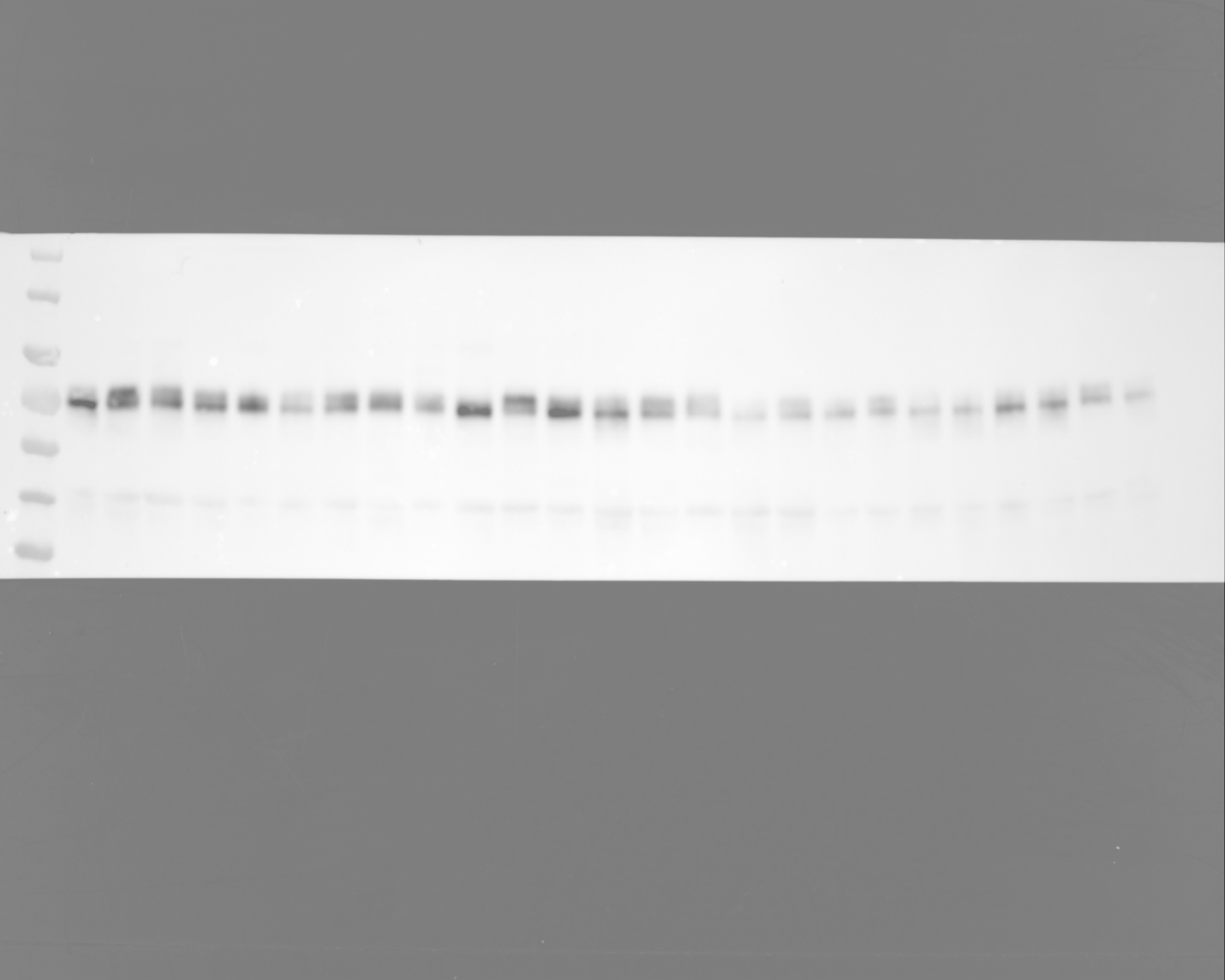


**Figure 1-source data 2**:

Normalizing ponceau stain whole blot for JPh1 blot for figure 1A


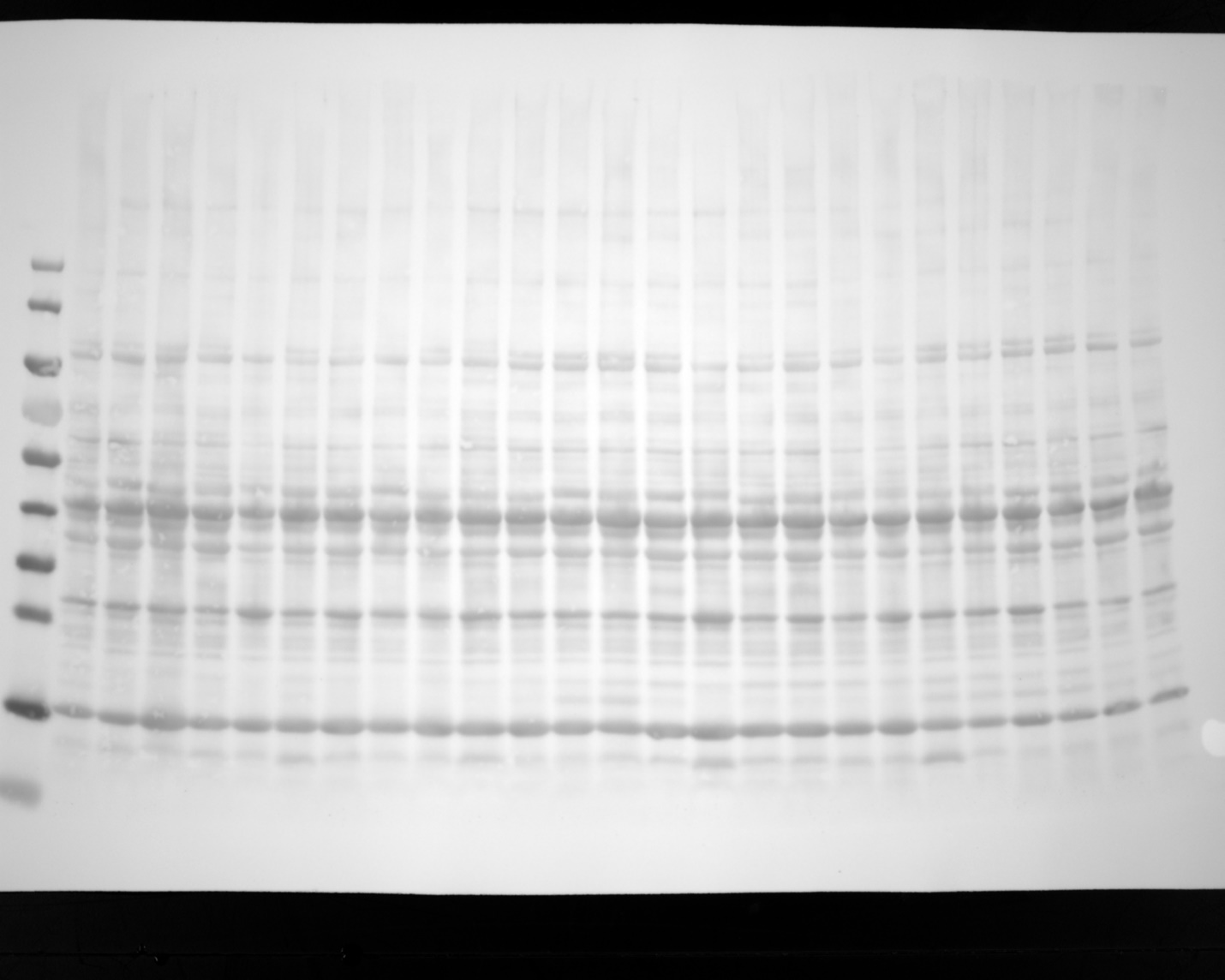


**Figure 1-source data 3**: JPh44 raw blot shown in Fig 1B

MHN MHS


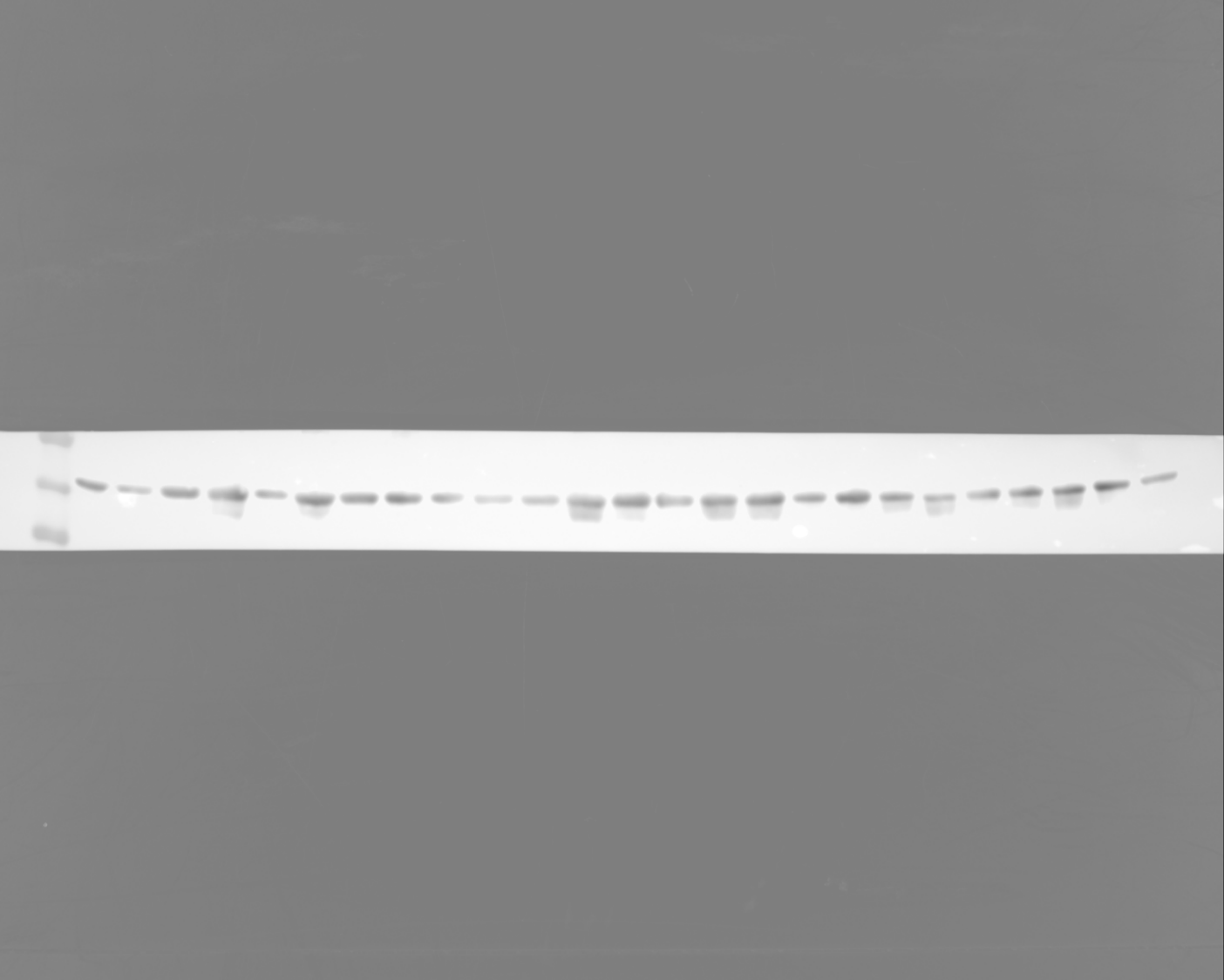


**Figure 1-source data 4:** Normalizing ponceau stain whole blot for JPh44 blot of figure 1B


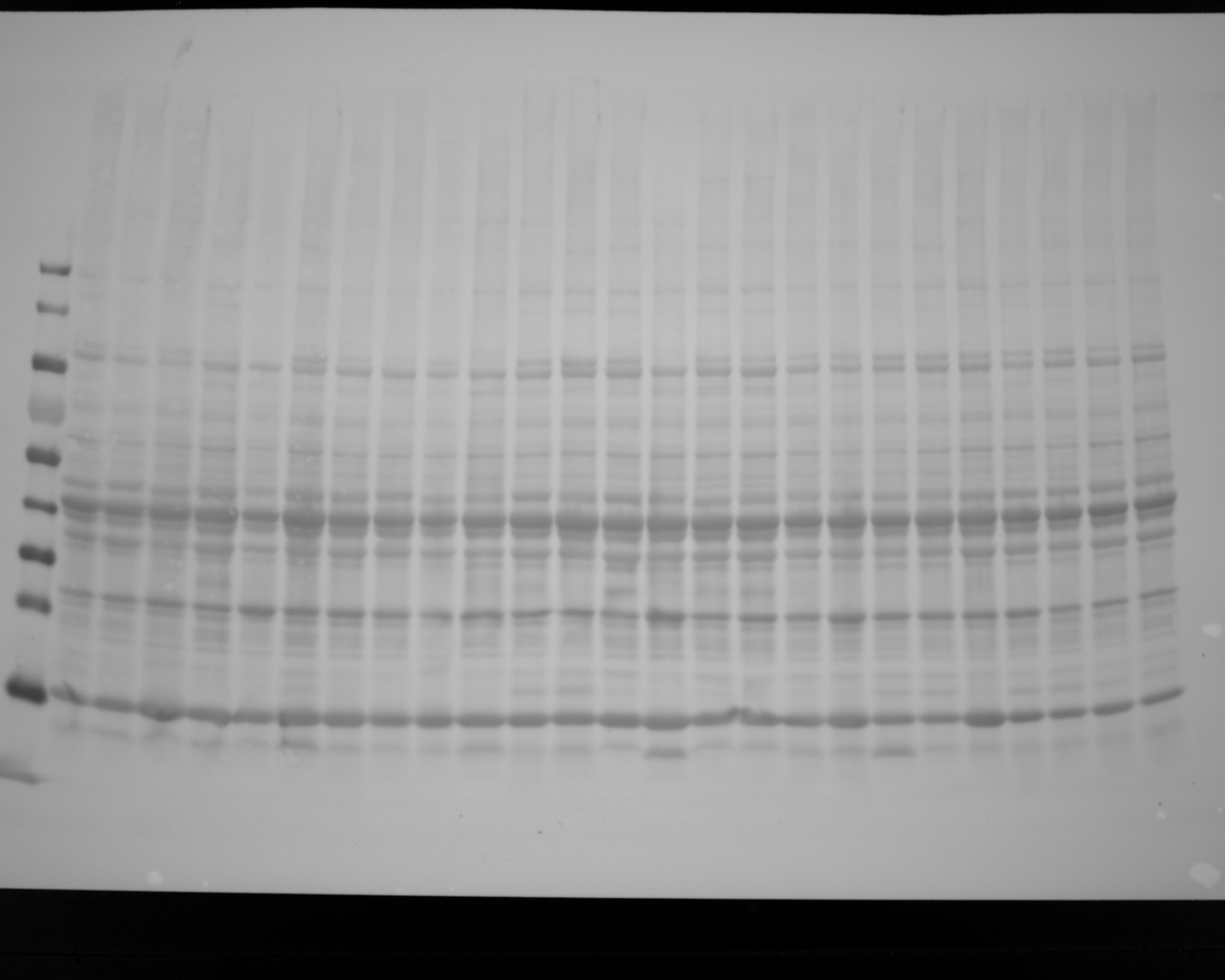


**Figure 1-source data 5**: GSK3b raw blot shown in Fig 1F

MHN MHS


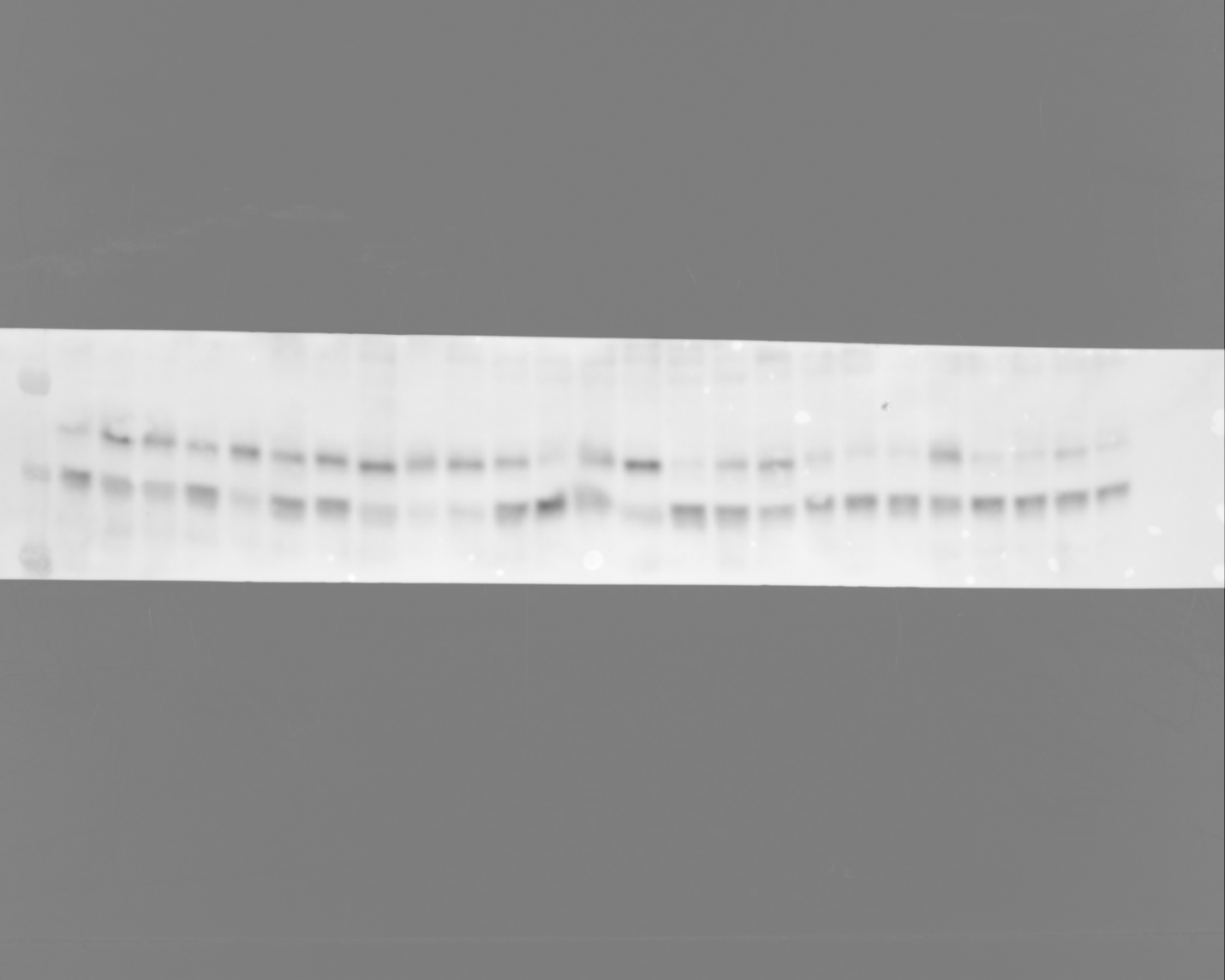


**Figure 1-source data 6**: Normalizing ponceau stain whole blot for GSK3B blot of figure 1f


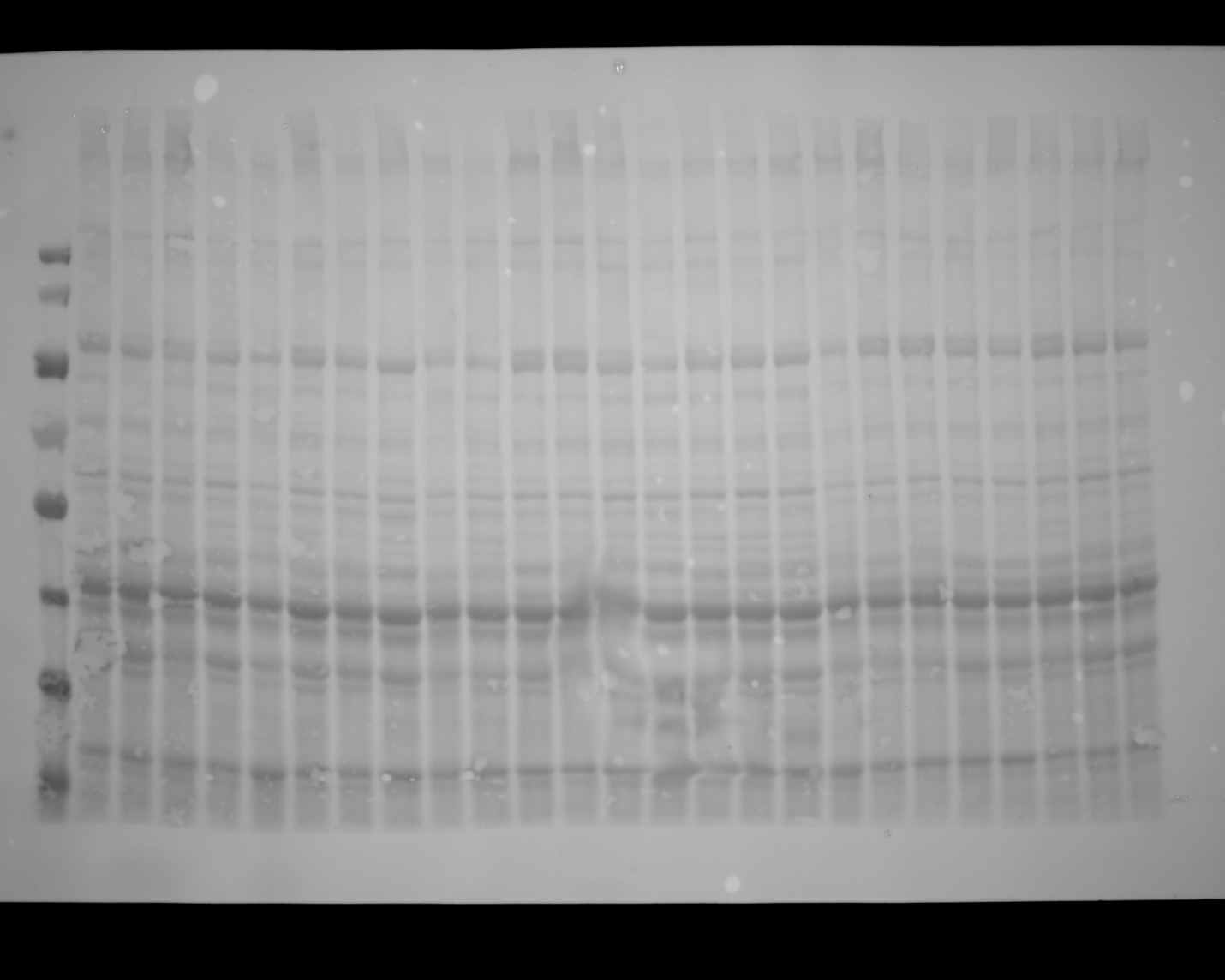

Supplement: Figure 1—source data 1. [file elife-78874-fig1-data1.zip › Figure 1-source data 1/Figure 1 source data annotated.docx]
