# Supplementary figures and images for: Muscle calcium stress cleaves junctophilin1, unleashing a gene regulatory program predicted to correct glucose dysregulation
Source: eLife. 2023 Feb 1;12:e78874. doi: 10.7554/eLife.78874 (PMC9891728; doi:10.7554/eLife.78874)

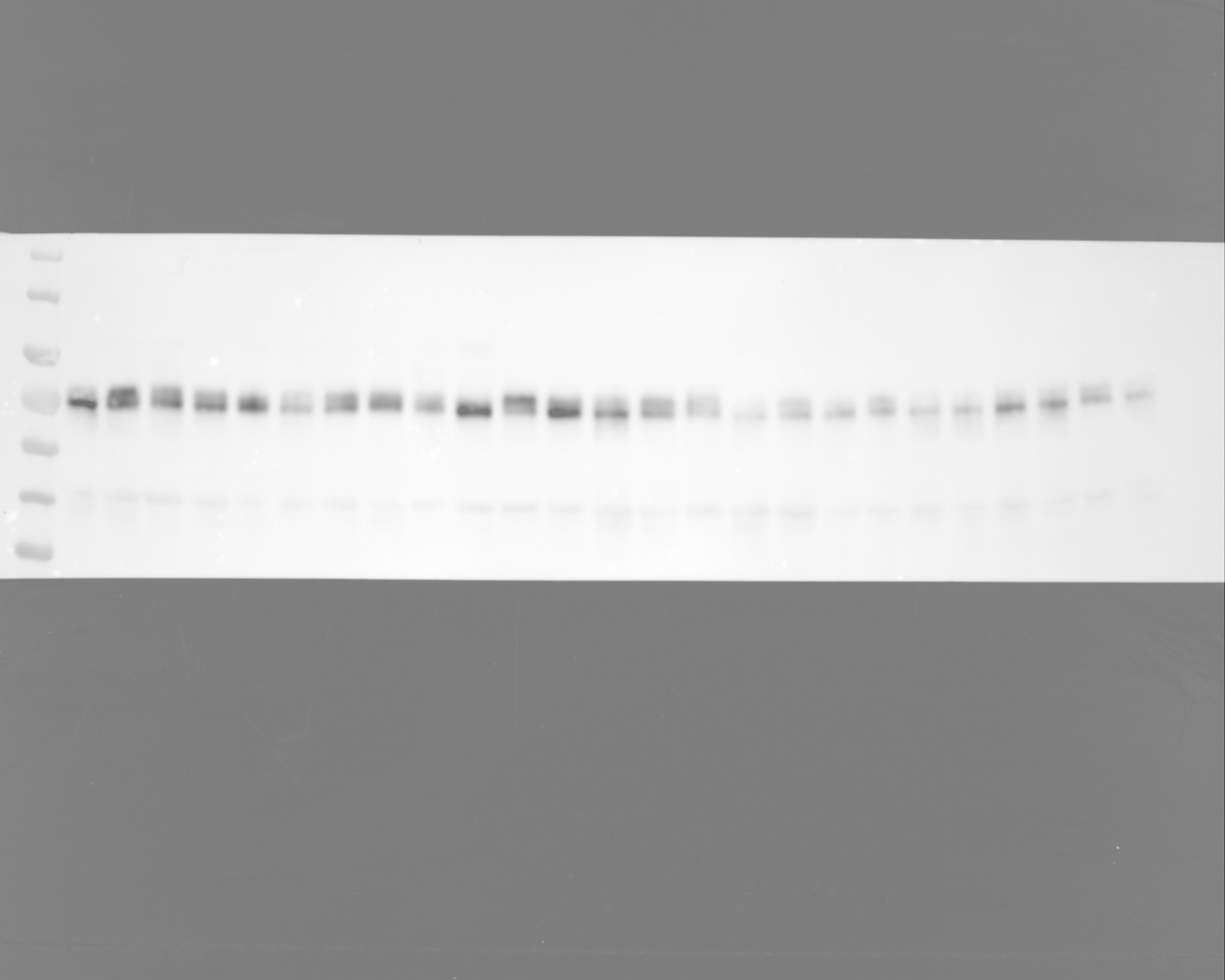

Supplement: Figure 1—source data 1. [file elife-78874-fig1-data1.zip › Figure 1-source data 1/Figure 1-source data 1.tif]

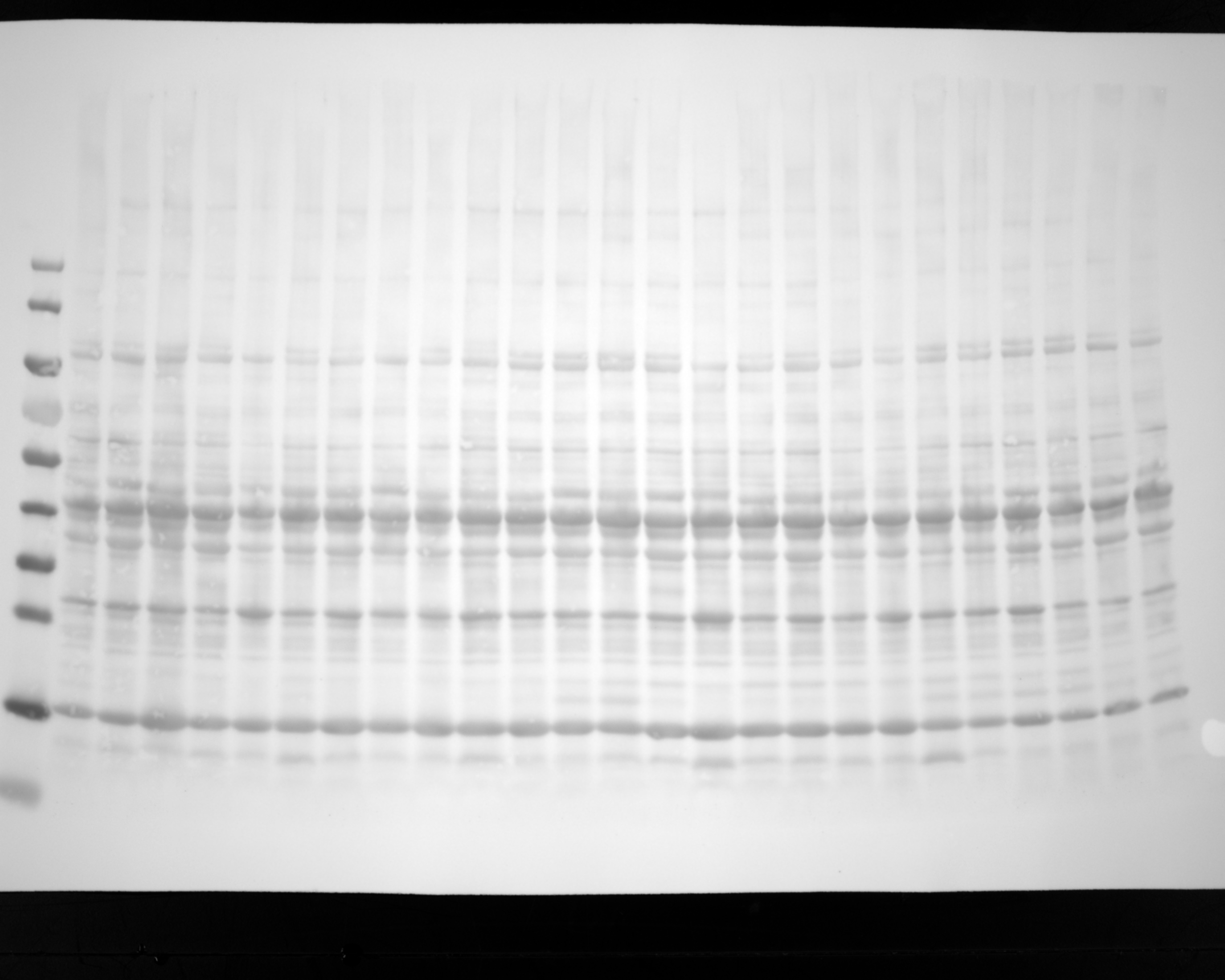

Supplement: Figure 1—source data 2. [file elife-78874-fig1-data2.zip › Figure 1-source data 2/Figure 1-source data 2.tif]

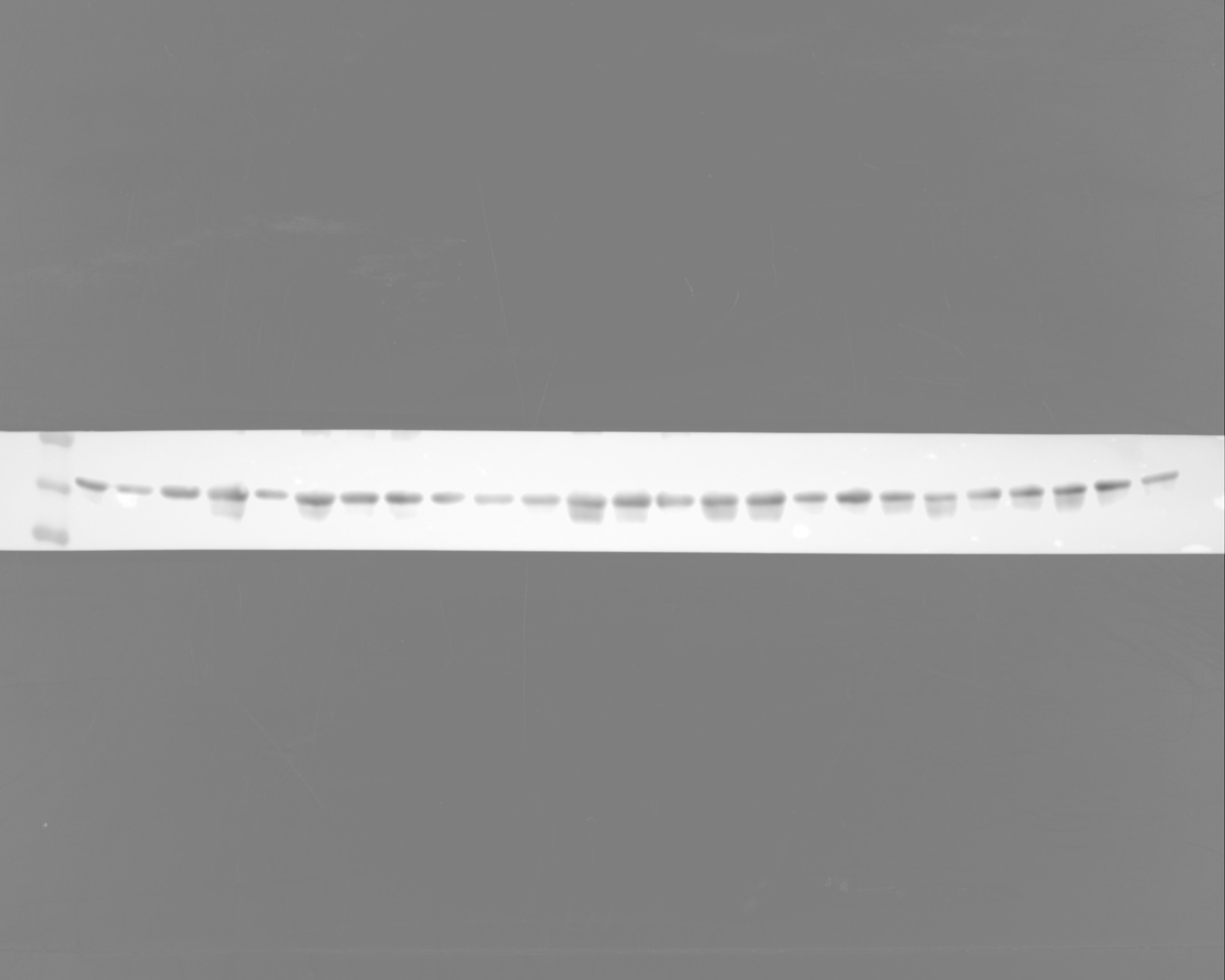

Supplement: Figure 1—source data 3. [file elife-78874-fig1-data3.zip › Figure 1-source data 3/Figure 1-source data 3.tif]

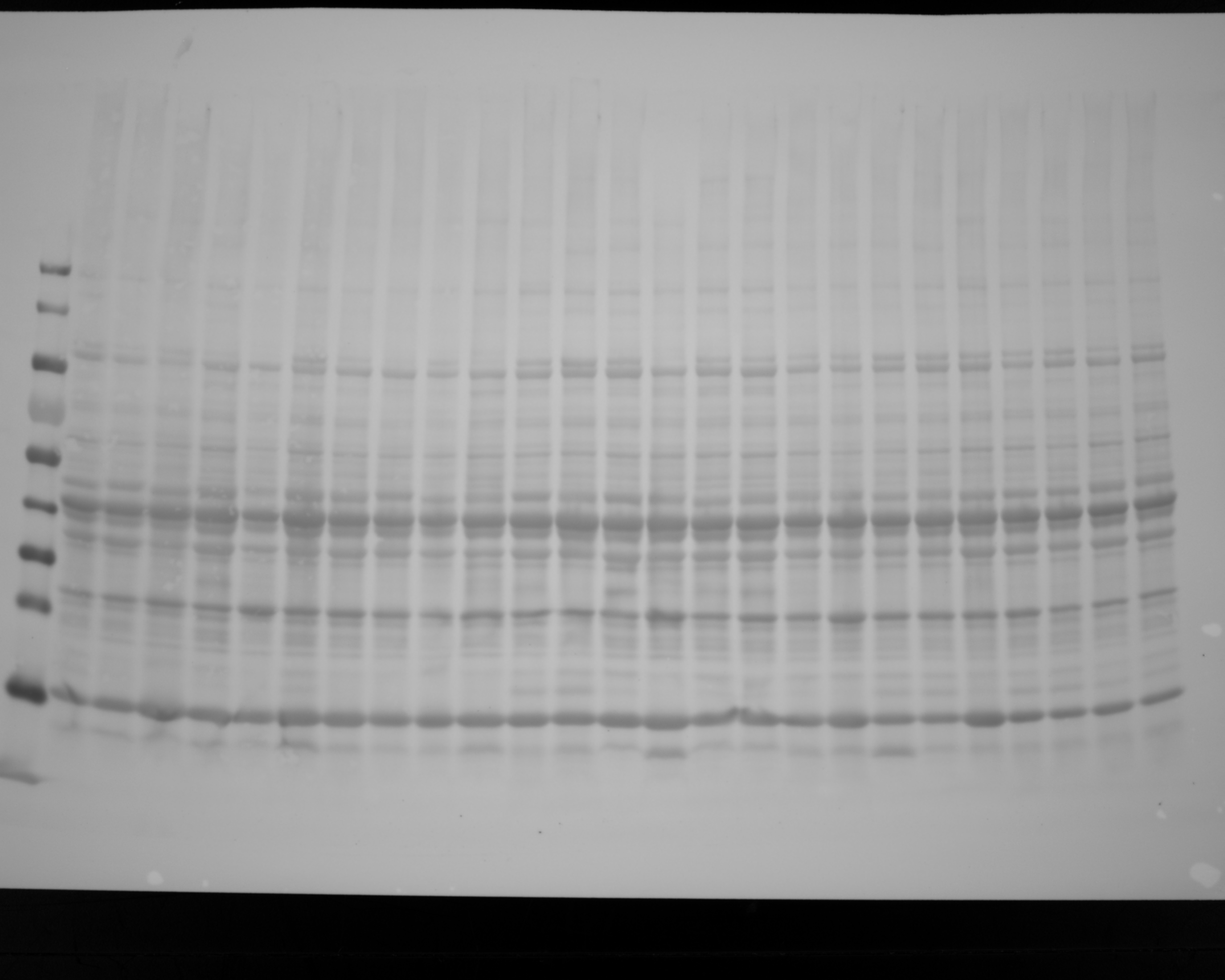

Supplement: Figure 1—source data 4. [file elife-78874-fig1-data4.zip › Figure 1-source data 4/Figure 1-source data 4.tif]

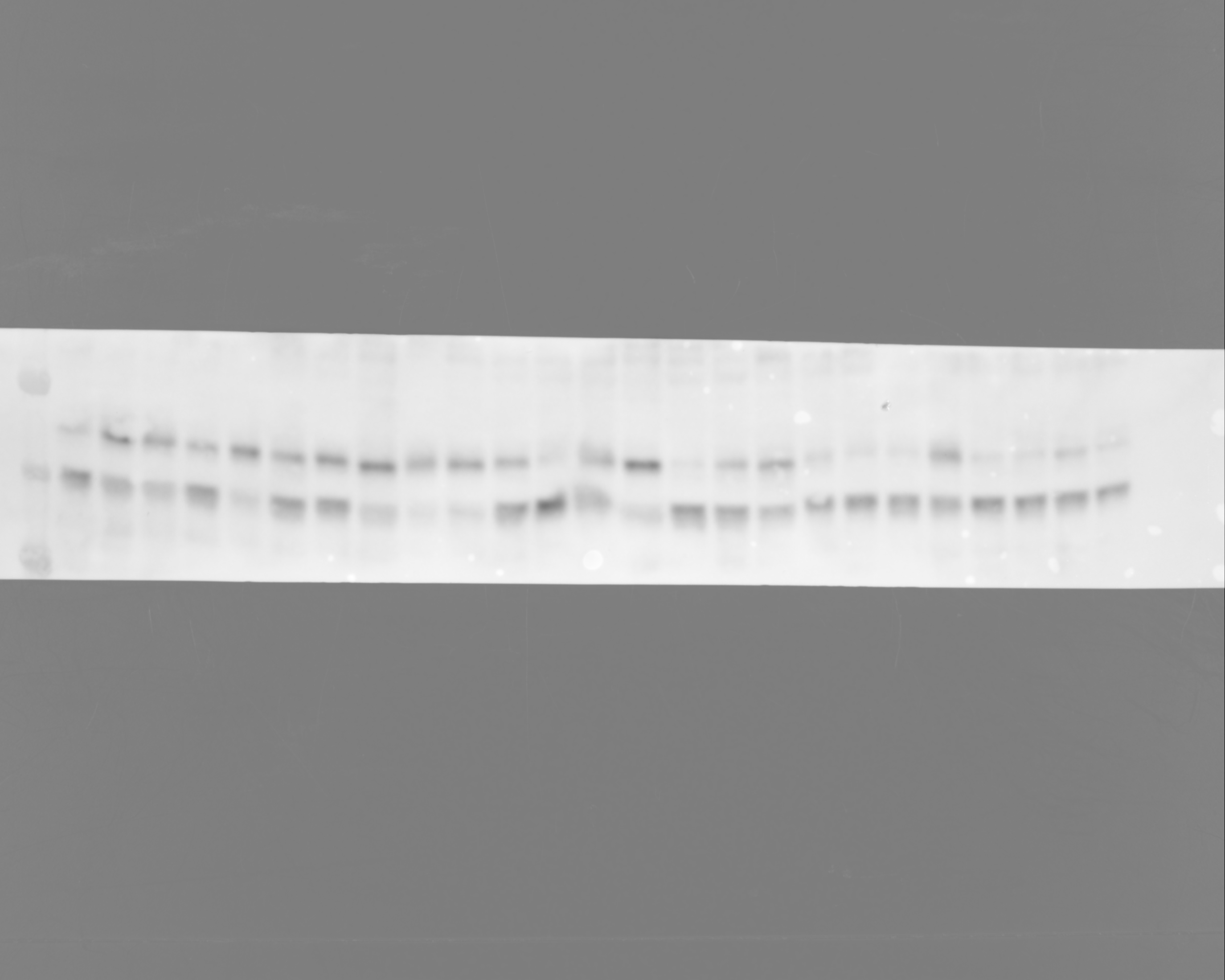

Supplement: Figure 1—source data 5. [file elife-78874-fig1-data5.zip › Figure 1-source data 5/Figure 1-source data 5 (1).tif]

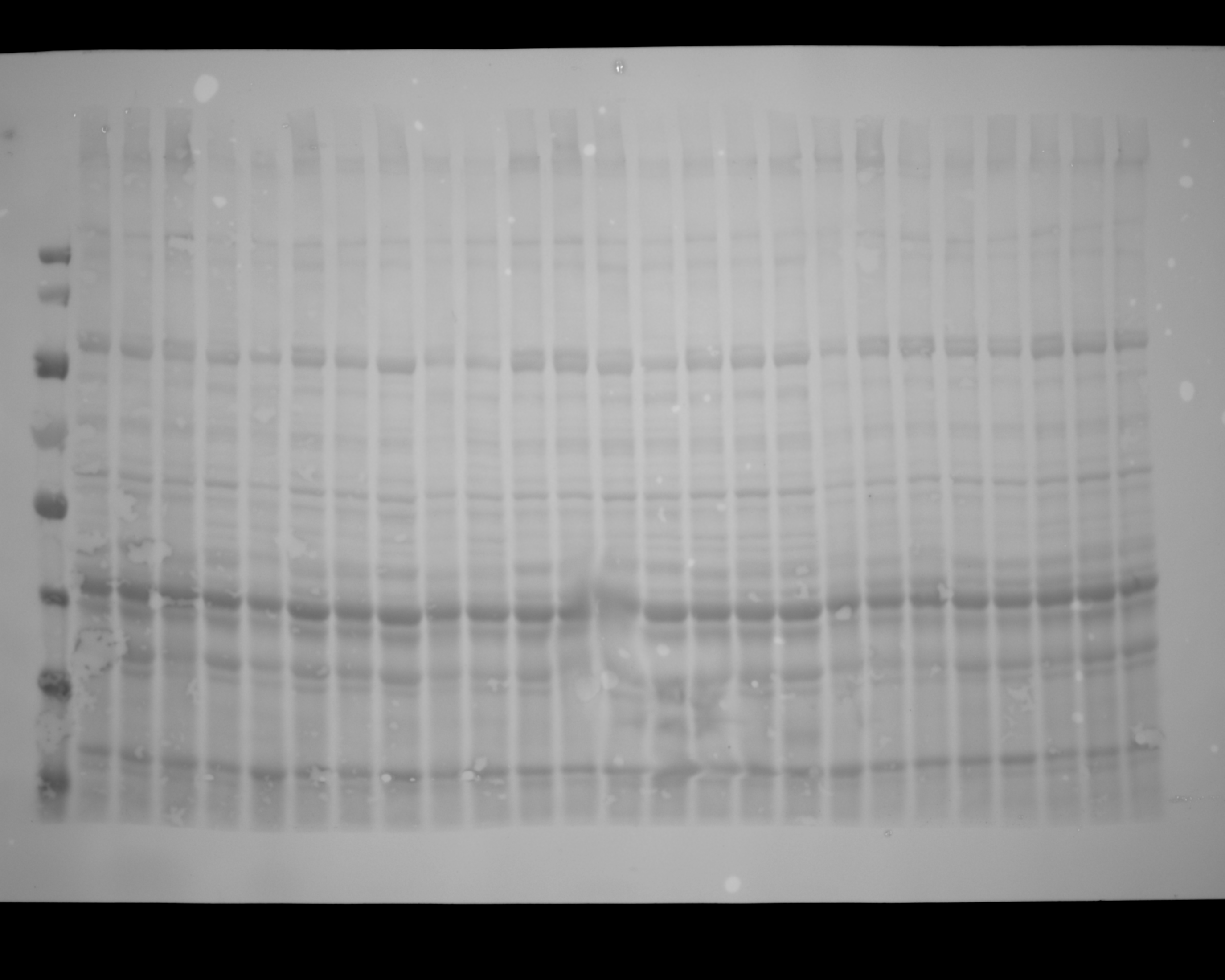

Supplement: Figure 1—source data 6. [file elife-78874-fig1-data6.zip › Figure 1-source data 6/Figure 1-source data 6.tif]

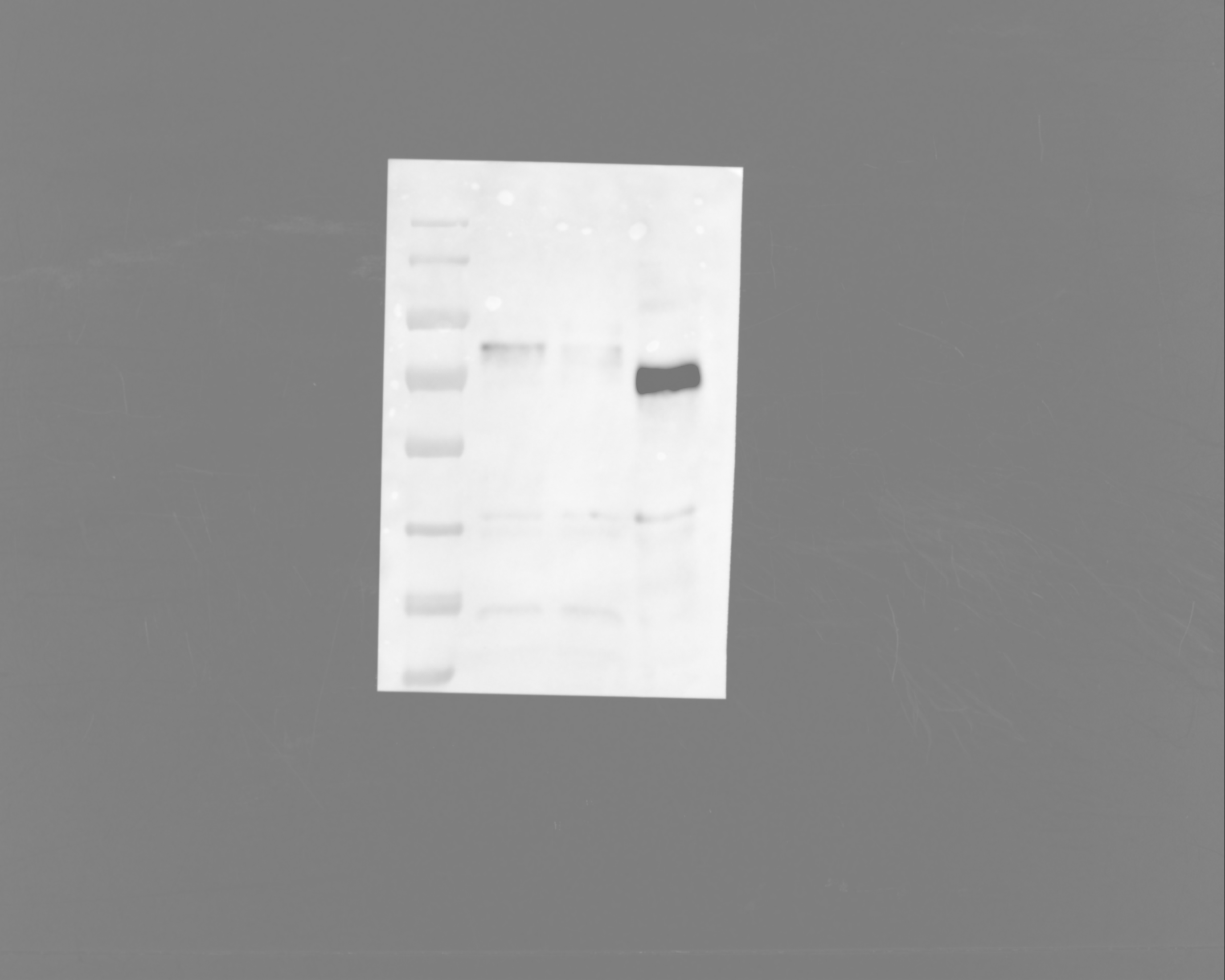

Supplement: Figure 1—figure supplement 1—source data 1. [file elife-78874-fig1-figsupp1-data1.zip › Figure 1-figure supplement 1-source data 1/Figure 1-figure supplement 1-source data 1.tif]

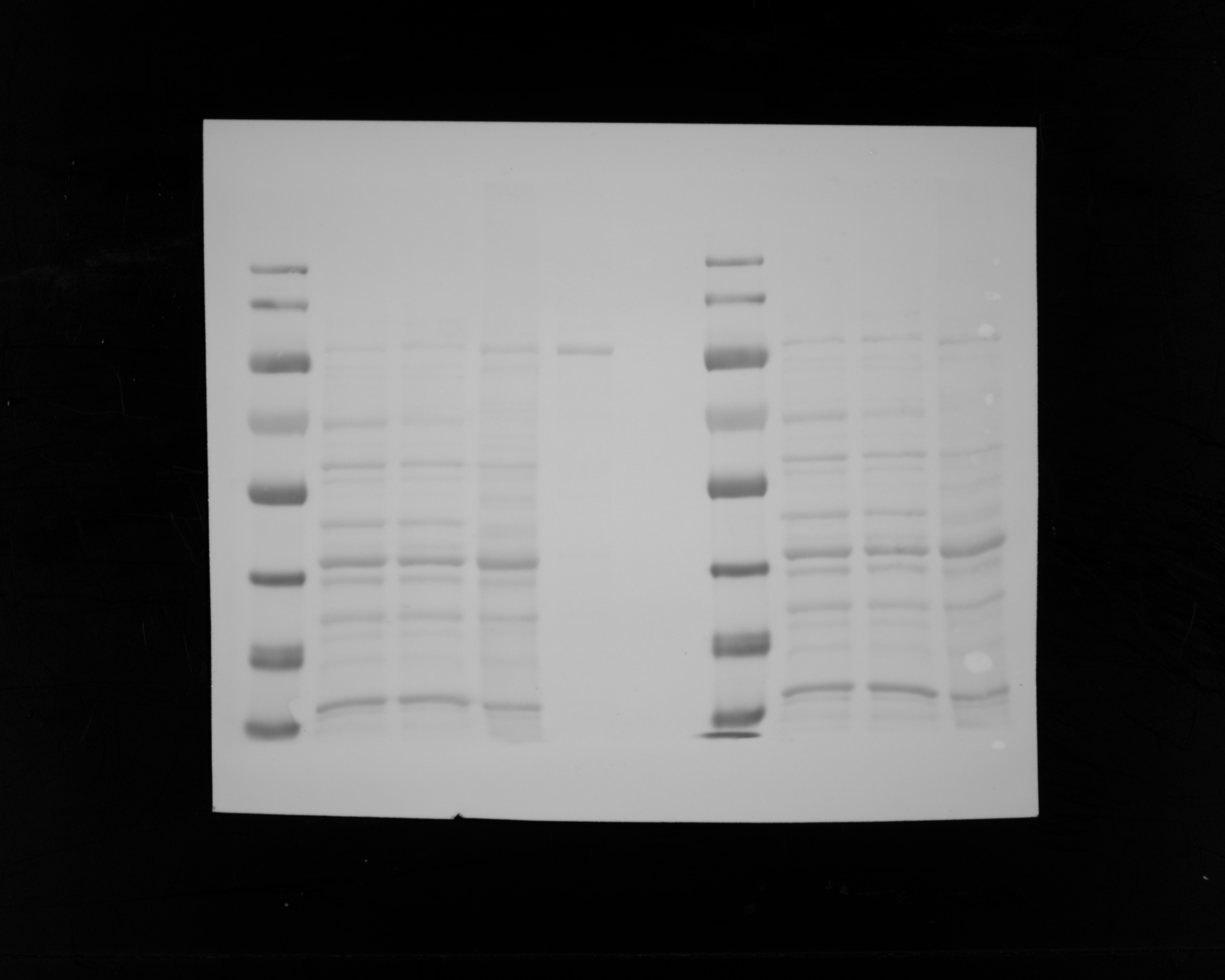

Supplement: Figure 1—figure supplement 1—source data 2. [file elife-78874-fig1-figsupp1-data2.zip › Figure 1-figure supplement 1-source data 2/Figure 1-figure supplement 1-source data 2.tif]

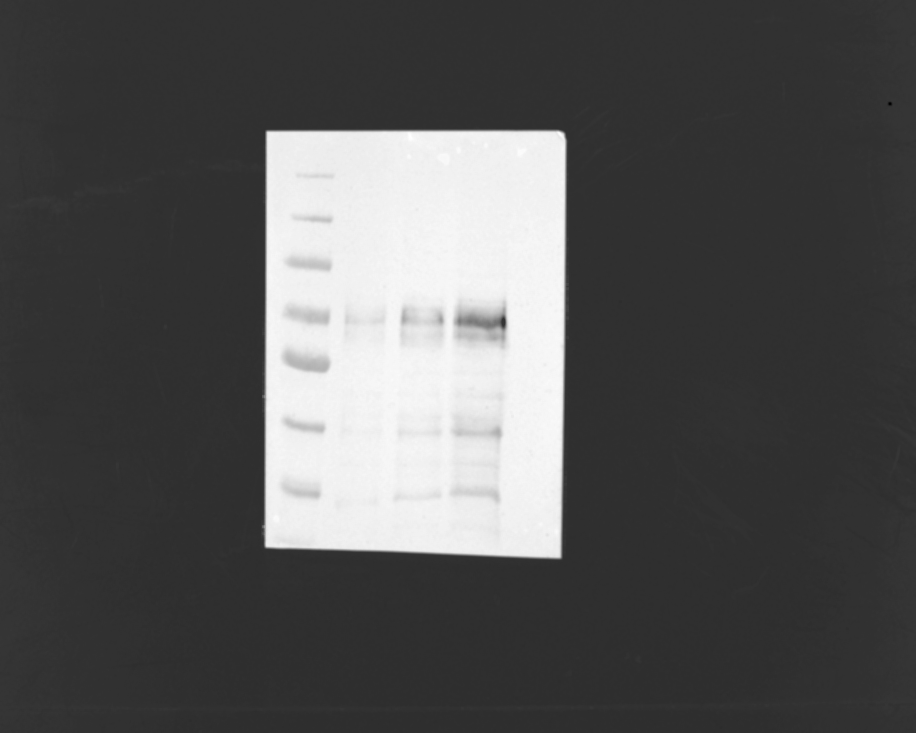

Supplement: Figure 1—figure supplement 2—source data 1. [file elife-78874-fig1-figsupp2-data1.zip › Figure 1-figure supplement 2-source data 1/Figure 1-figure supplement 2-source data 1.tif]

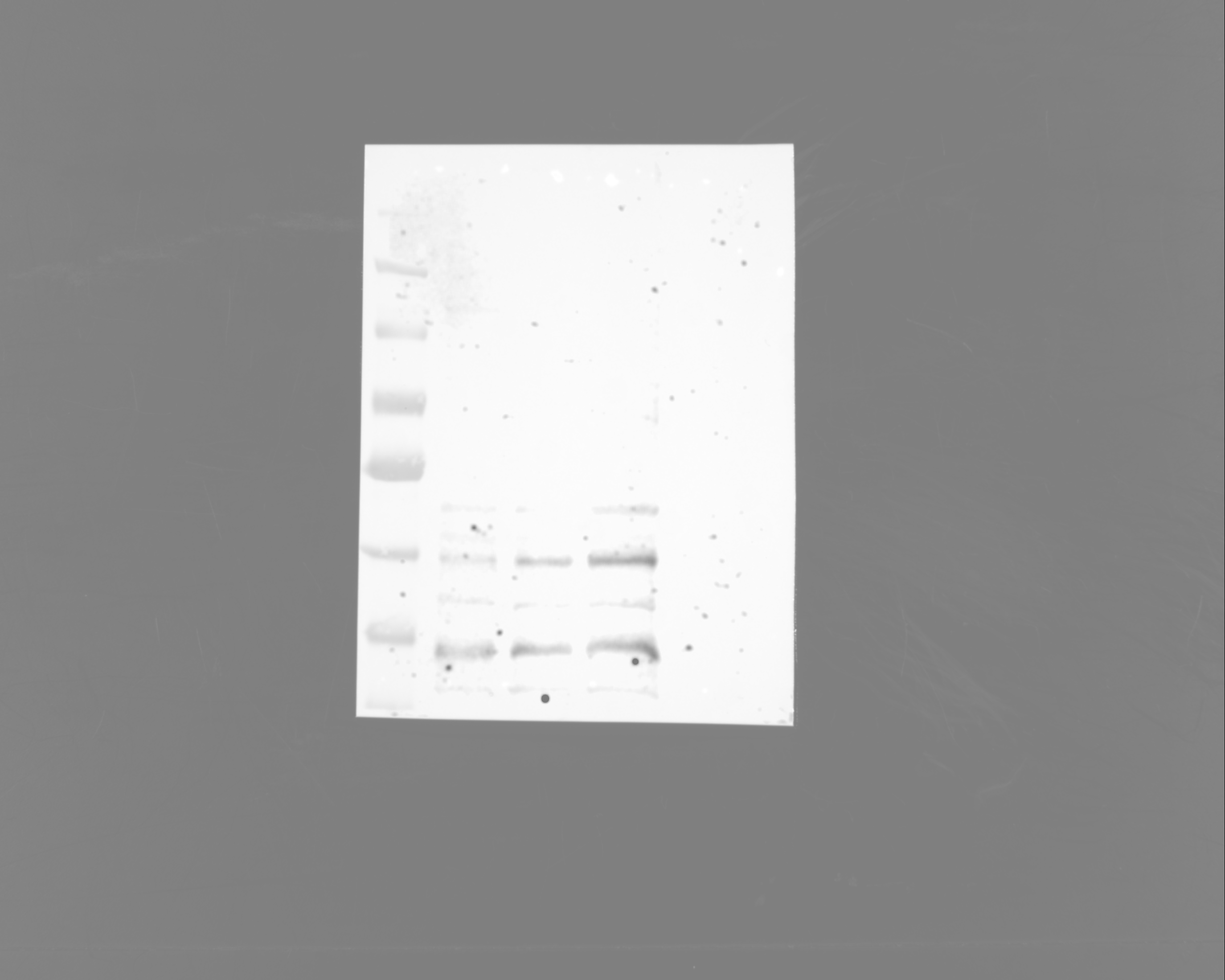

Supplement: Figure 1—figure supplement 2—source data 2. [file elife-78874-fig1-figsupp2-data2.zip › Figure 1-figure supplement 2-source data 2/Figure 1-figure supplement 2-source data 2.tif]

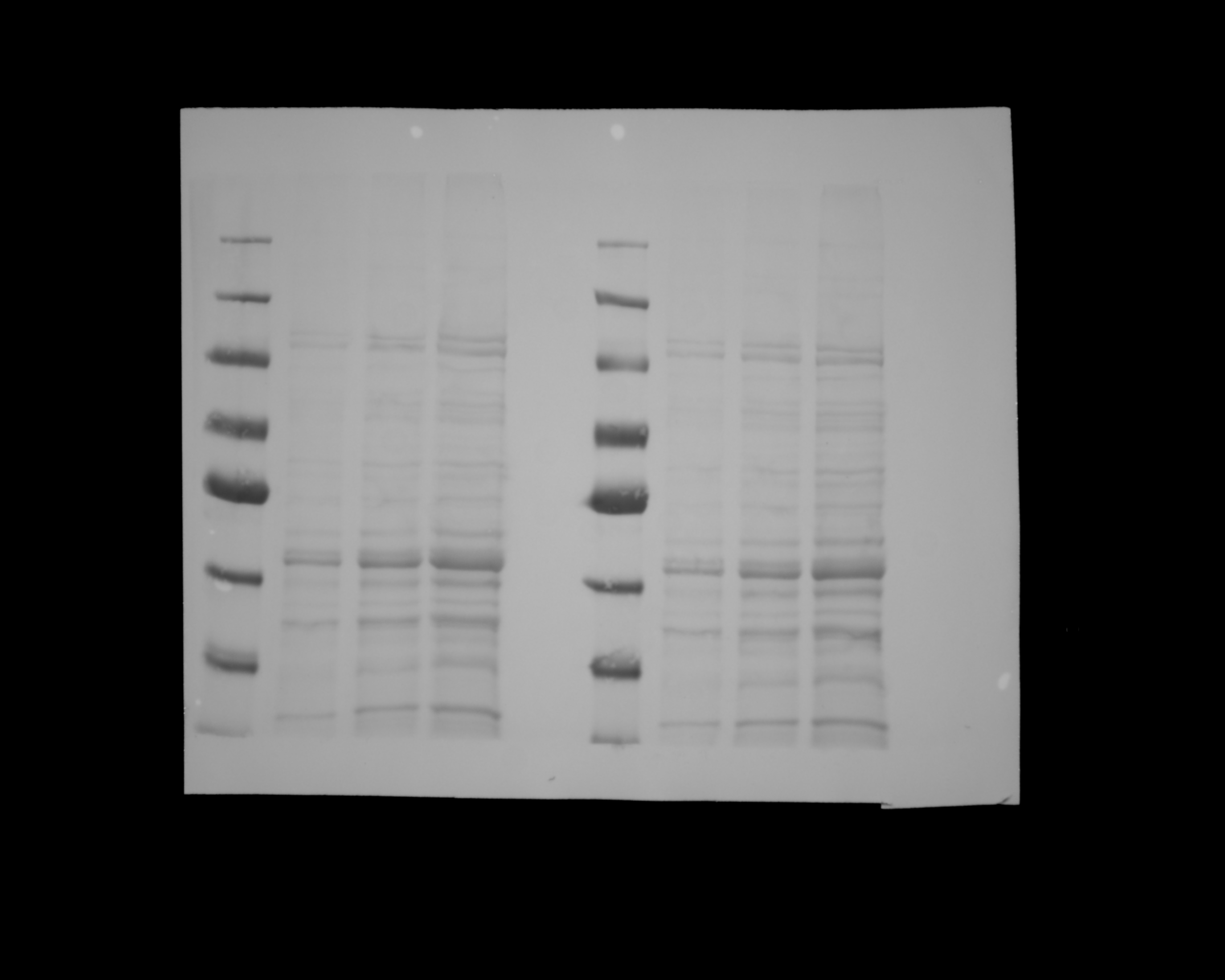

Supplement: Figure 1—figure supplement 2—source data 3. — Junctophilin abA blot is obtained from the left half and abB blot is obtained from the right half of the membrane shown. [file elife-78874-fig1-figsupp2-data3.zip › Figure 1-figure supplement 2-source data 3/Figure 1-figure supplement 2-source data 3.tif]

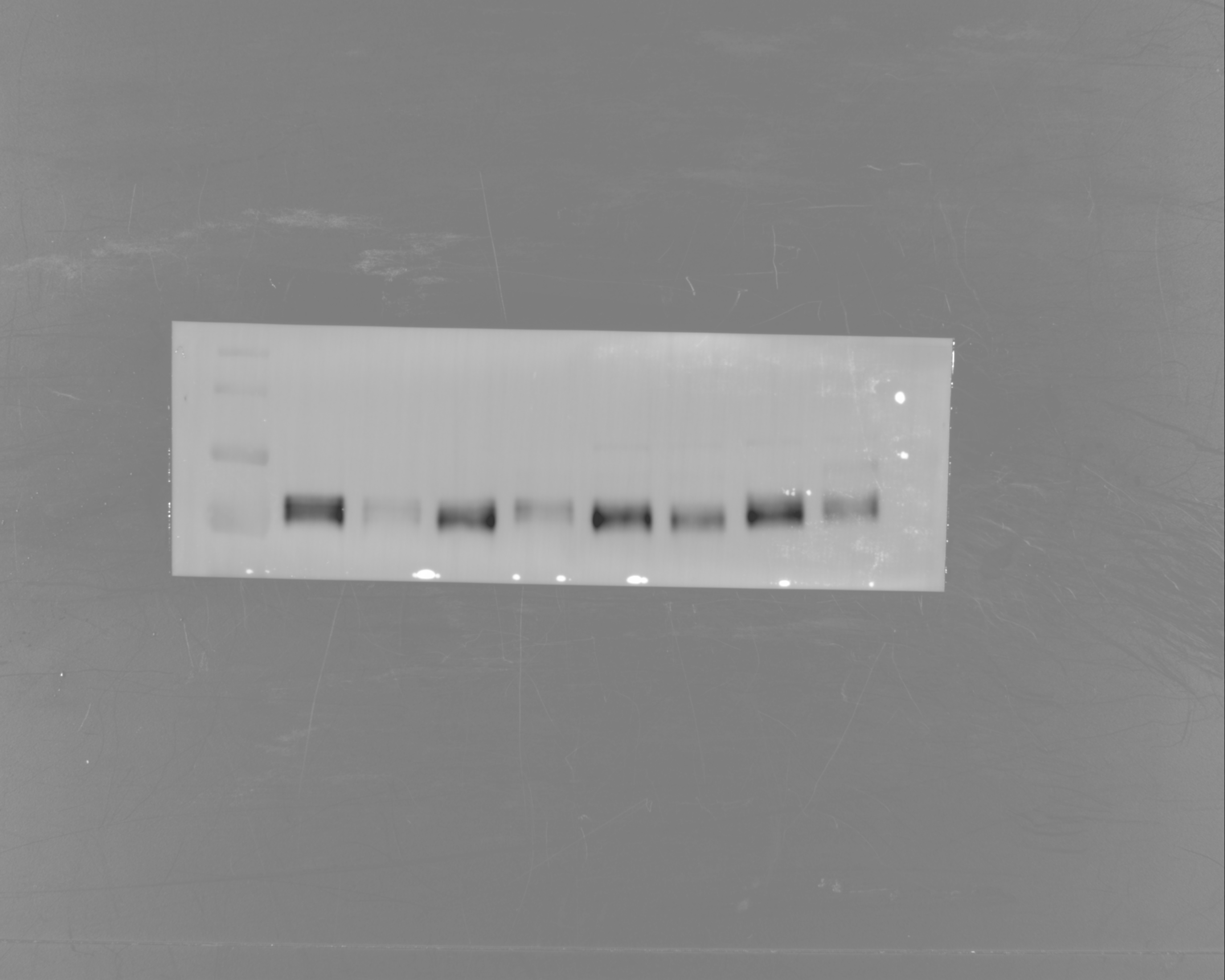

Supplement: Figure 3—source data 1. [file elife-78874-fig3-data1.zip › Figure 3-source data 1/Figure 3-source data 1.tif]

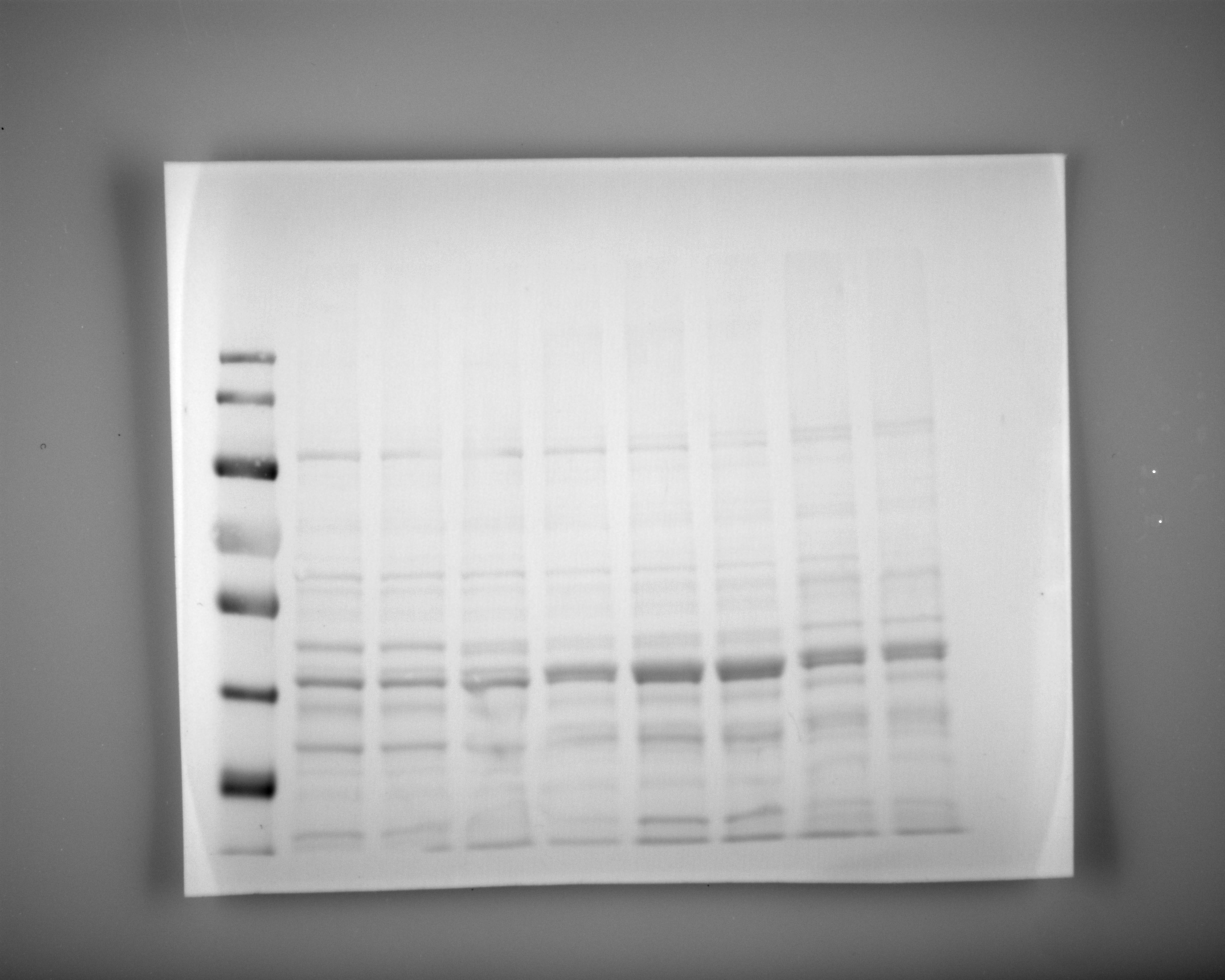

Supplement: Figure 3—source data 2. [file elife-78874-fig3-data2.zip › Figure 3-source data 2/Figure 3-source data 2.tif]

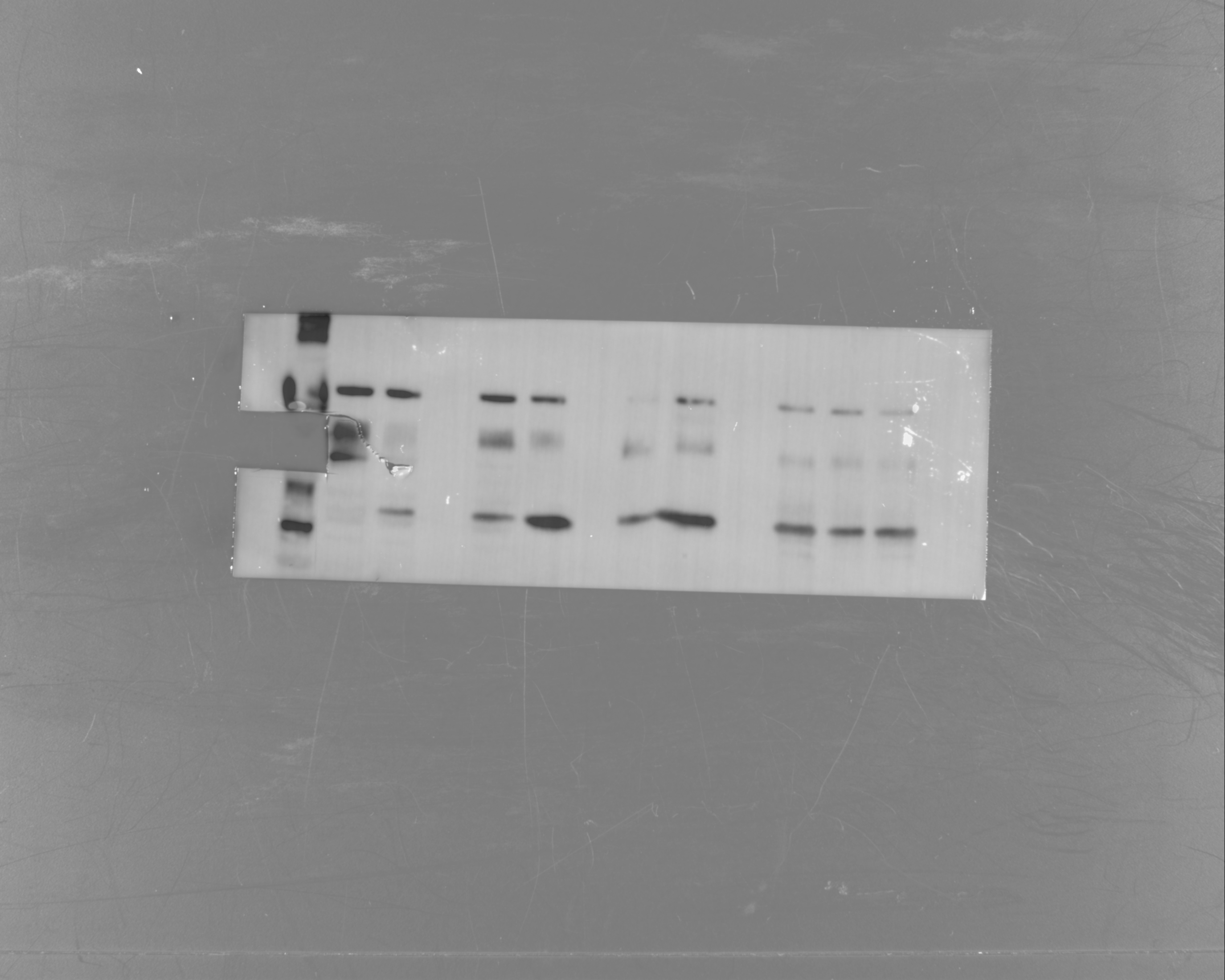

Supplement: Figure 3—source data 3. — The bands of upper molecular weight (particularly at 70 kDa) result from earlier incubation of same membrane with JPh1 abA and its incomplete stripping. Last 3 lanes in the blot are unrelated to the experiment. [file elife-78874-fig3-data3.zip › Figure 3-source data 3/Figure 3-source data 3.tif]

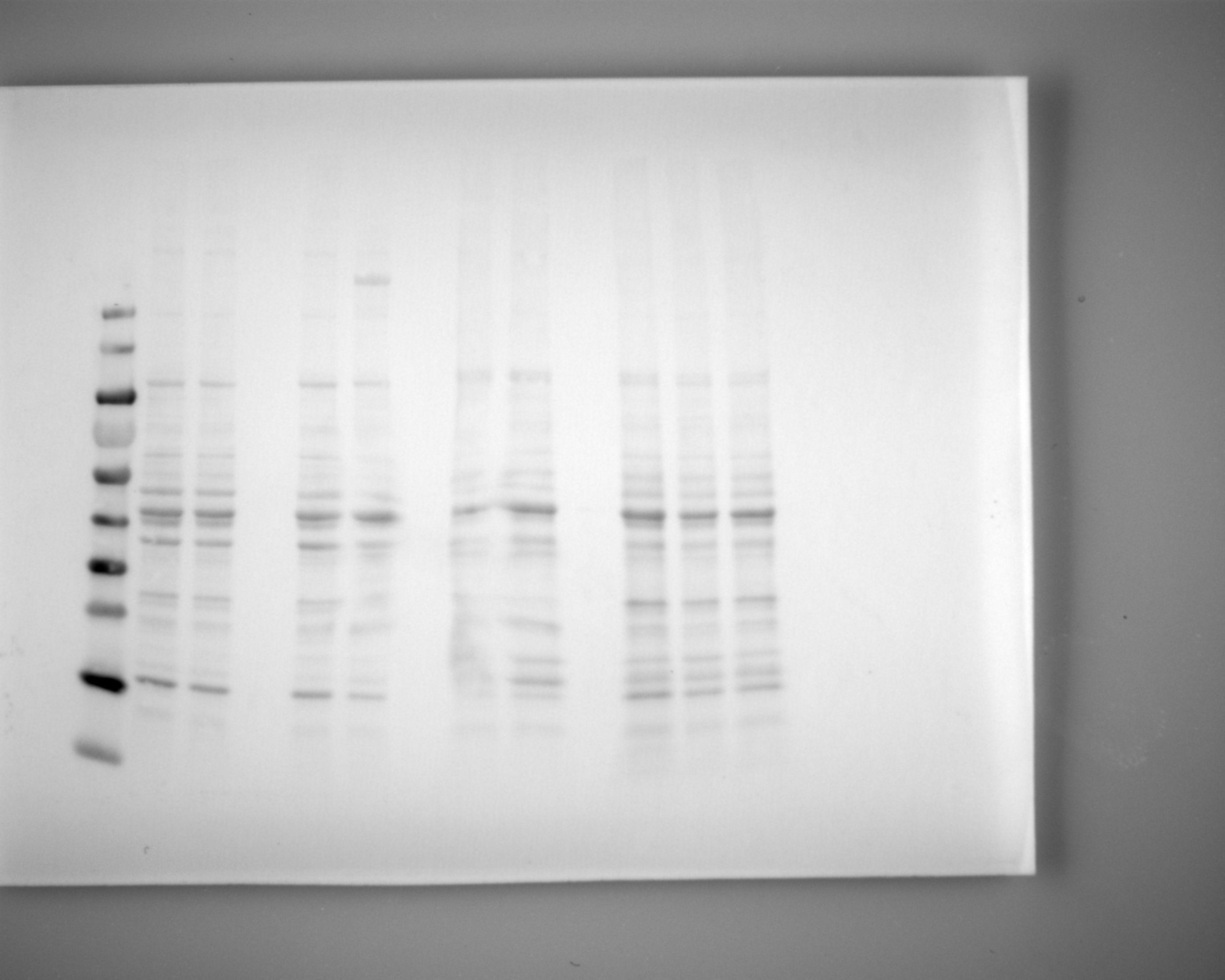

Supplement: Figure 3—source data 4. [file elife-78874-fig3-data4.zip › Figure 3-source data 4/Figure 3-source data 4.tif]

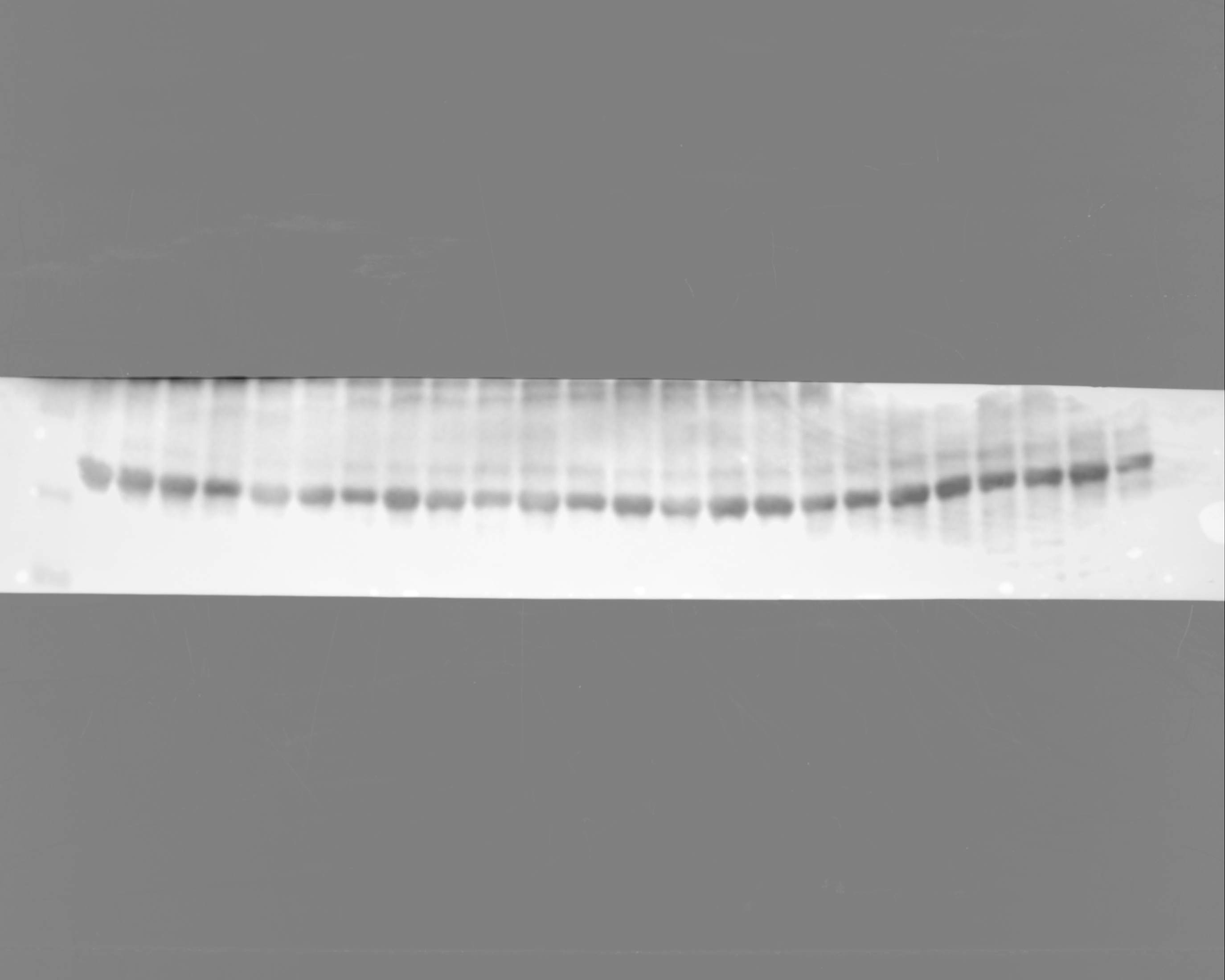

Supplement: Figure 3—source data 5. [file elife-78874-fig3-data5.zip › Figure 3-source data 5/Figure 3-source data 5.tif]

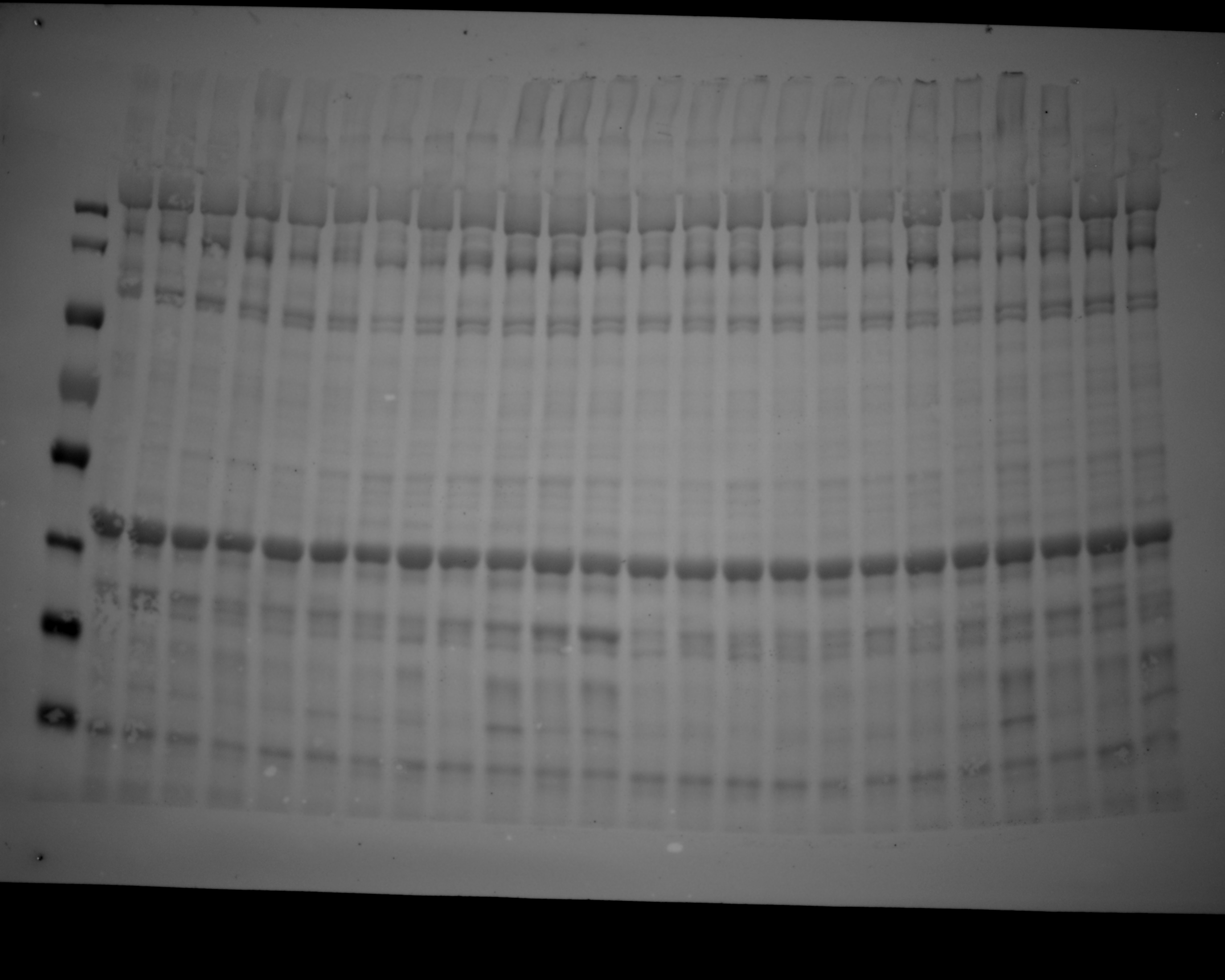

Supplement: Figure 3—source data 6. [file elife-78874-fig3-data6.zip › Figure 3-source data 6/Figure 3-source data 6.tif]

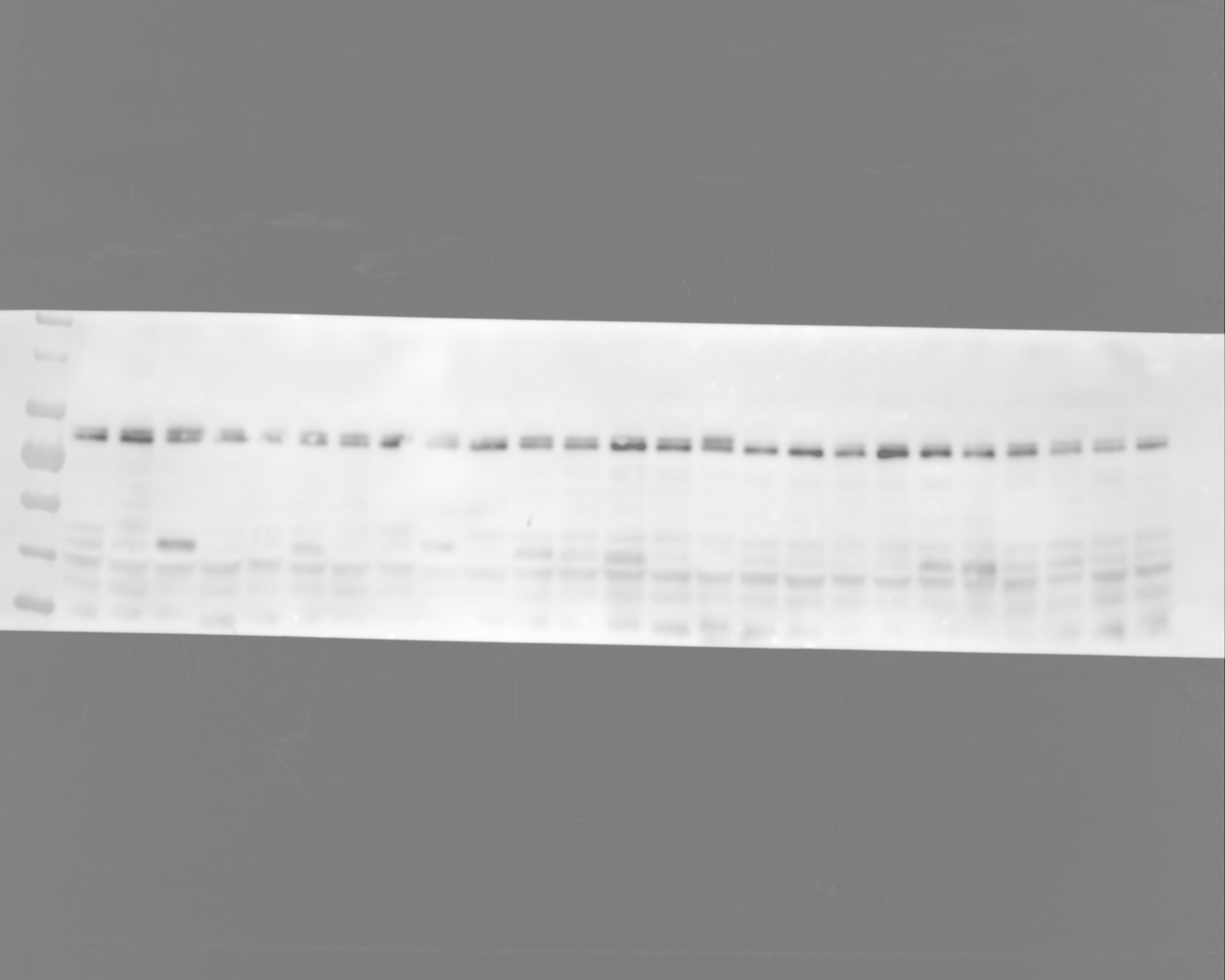

Supplement: Figure 4—source data 1. — Lower part of the blot, with calpain fragments of small molecular weight is shown in Figure 4—figure supplement 2. [file elife-78874-fig4-data1.zip › Figure 4- source data 1/Figure 4- source data 1.tif]

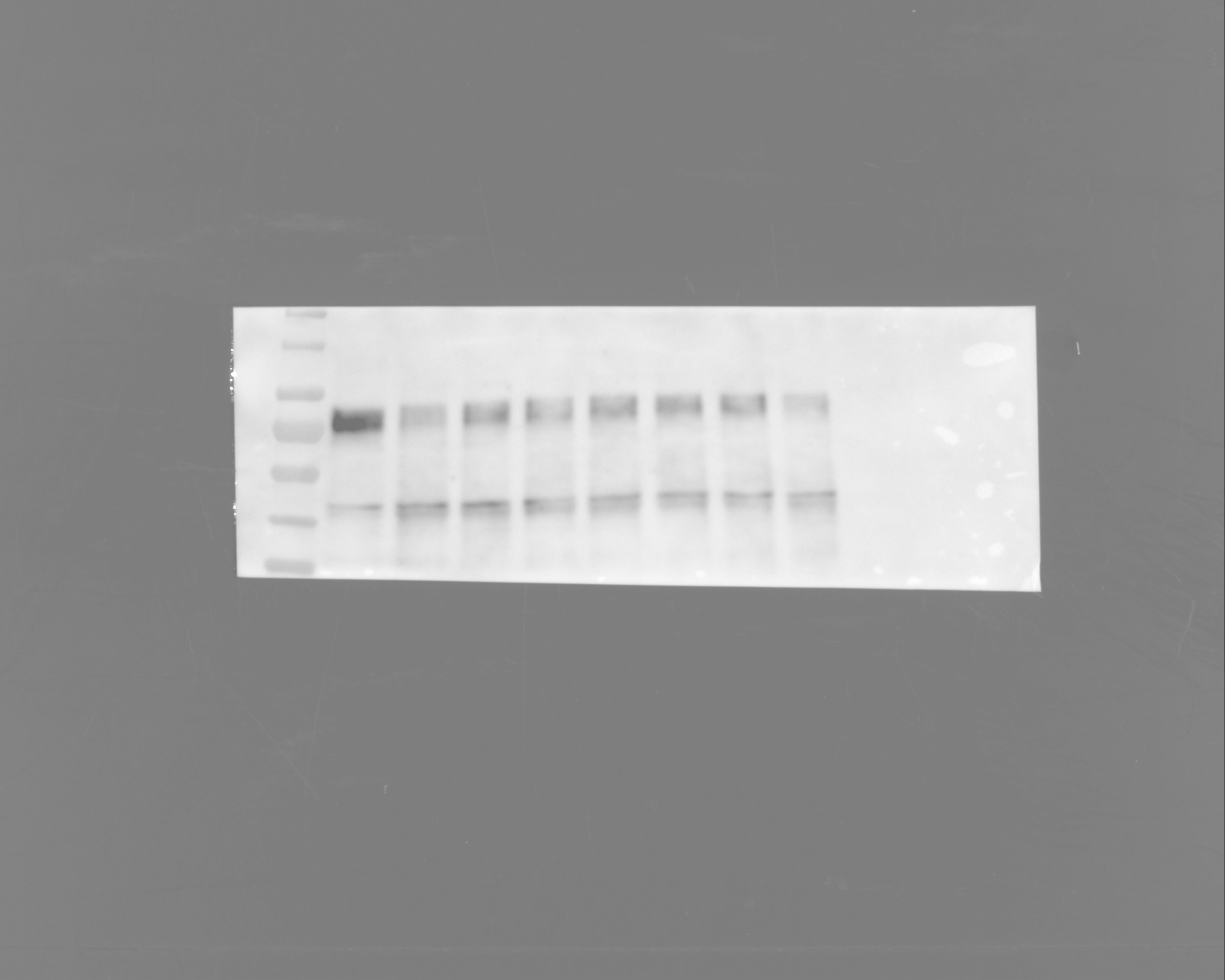

Supplement: Figure 5—source data 1. [file elife-78874-fig5-data1.zip › Figure 5-source data 1/Figure 5-source data 1.tif]

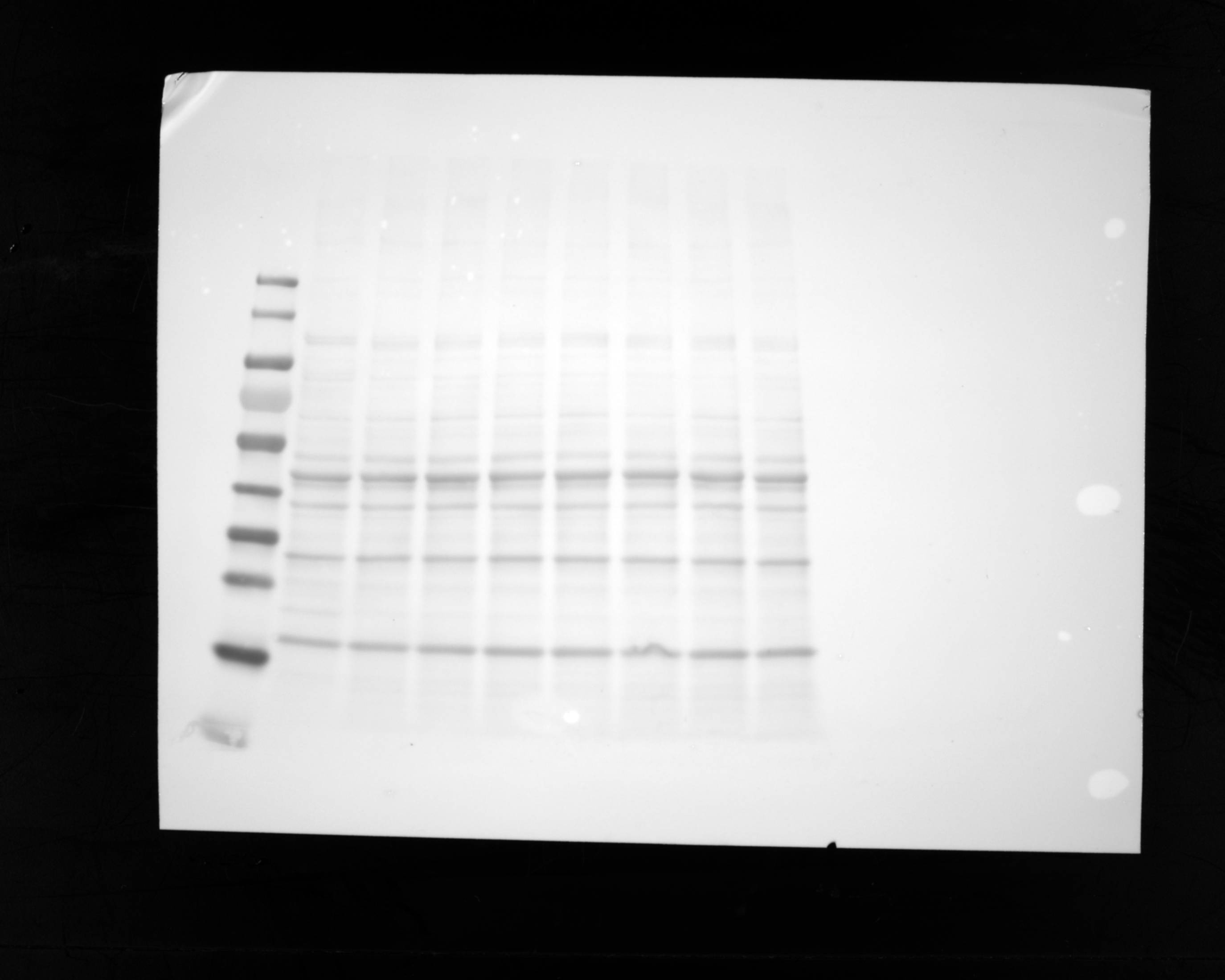

Supplement: Figure 5—source data 2. [file elife-78874-fig5-data2.zip › Figure 5-source data 2/Figure 5-source data 2.tif]

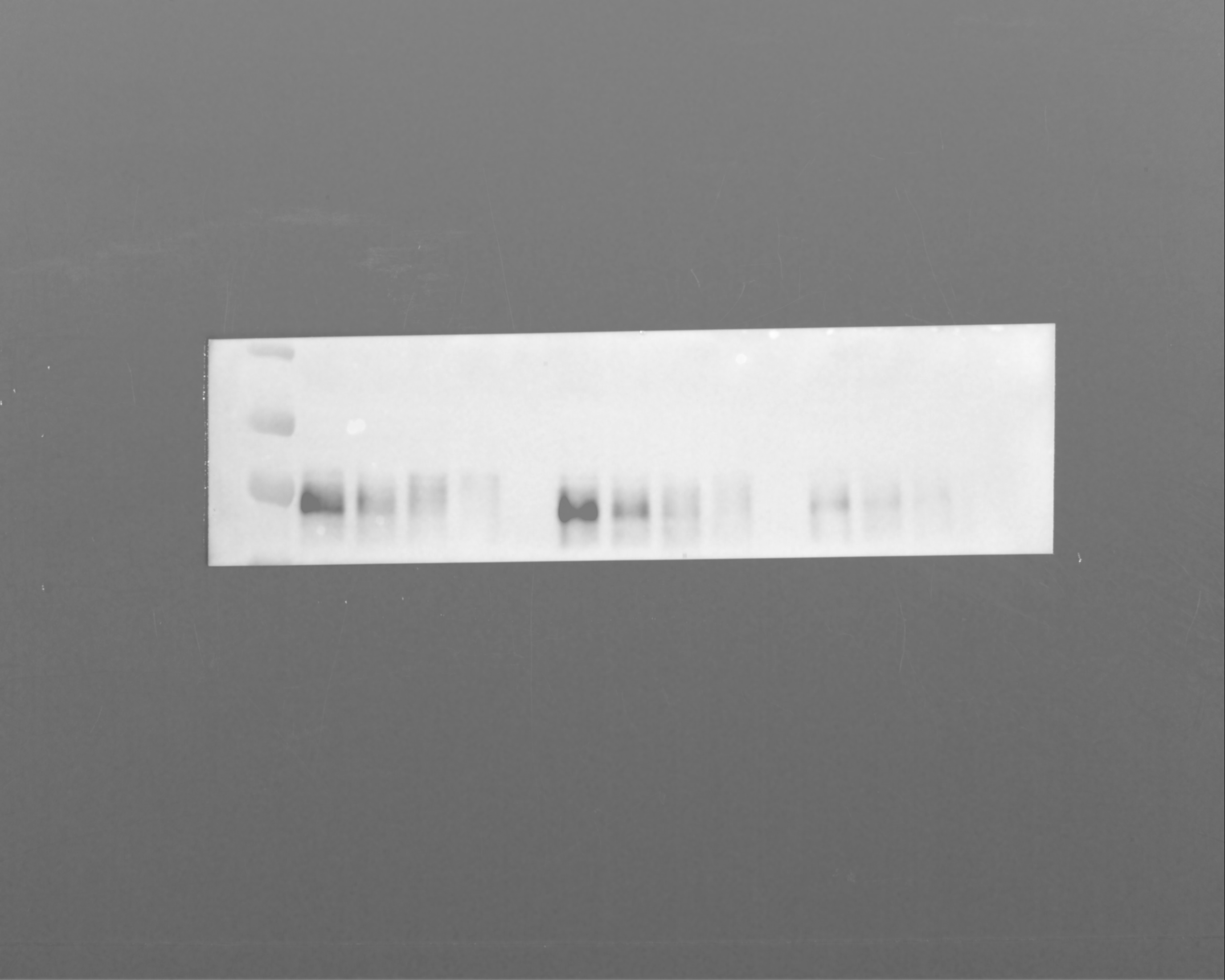

Supplement: Figure 5—source data 3. [file elife-78874-fig5-data3.zip › Figure 5-source data 3/Figure 5-source data 3.tif]

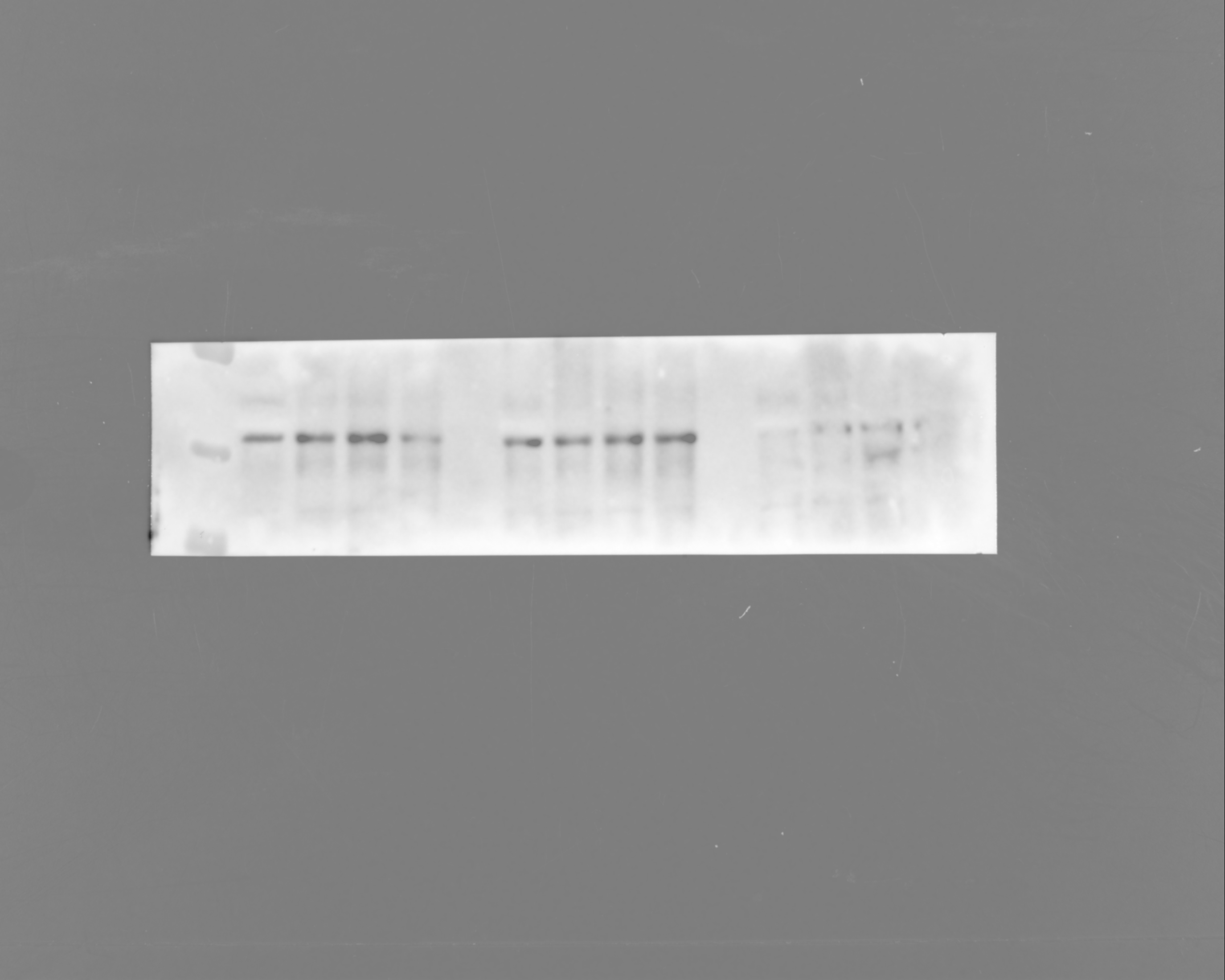

Supplement: Figure 5—source data 4. [file elife-78874-fig5-data4.zip › Figure 5-source data 4/Figure 5-source data 4.tif]

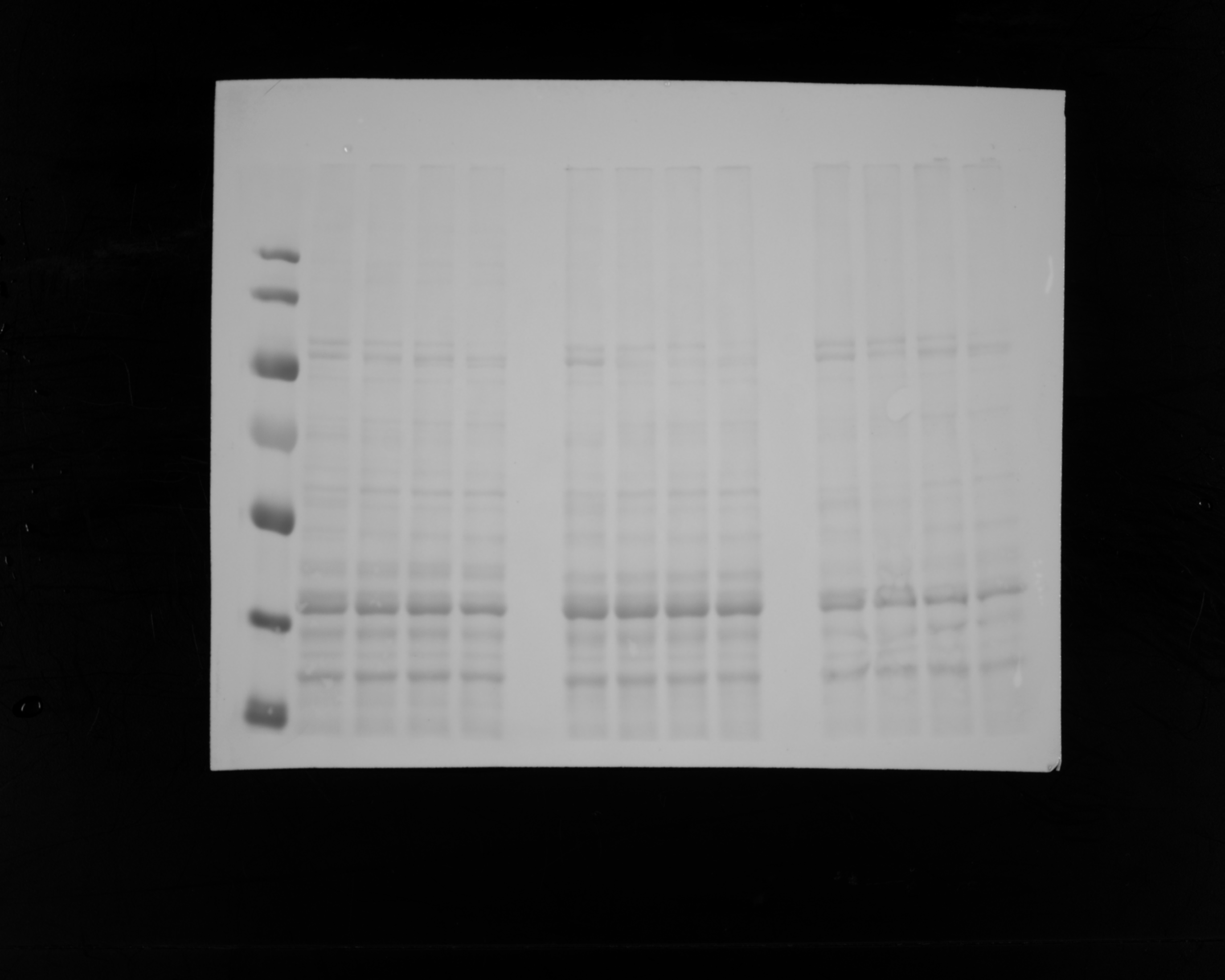

Supplement: Figure 5—source data 5. [file elife-78874-fig5-data5.zip › Figure 5-source data 5/Figure 5-source data 5.tif]

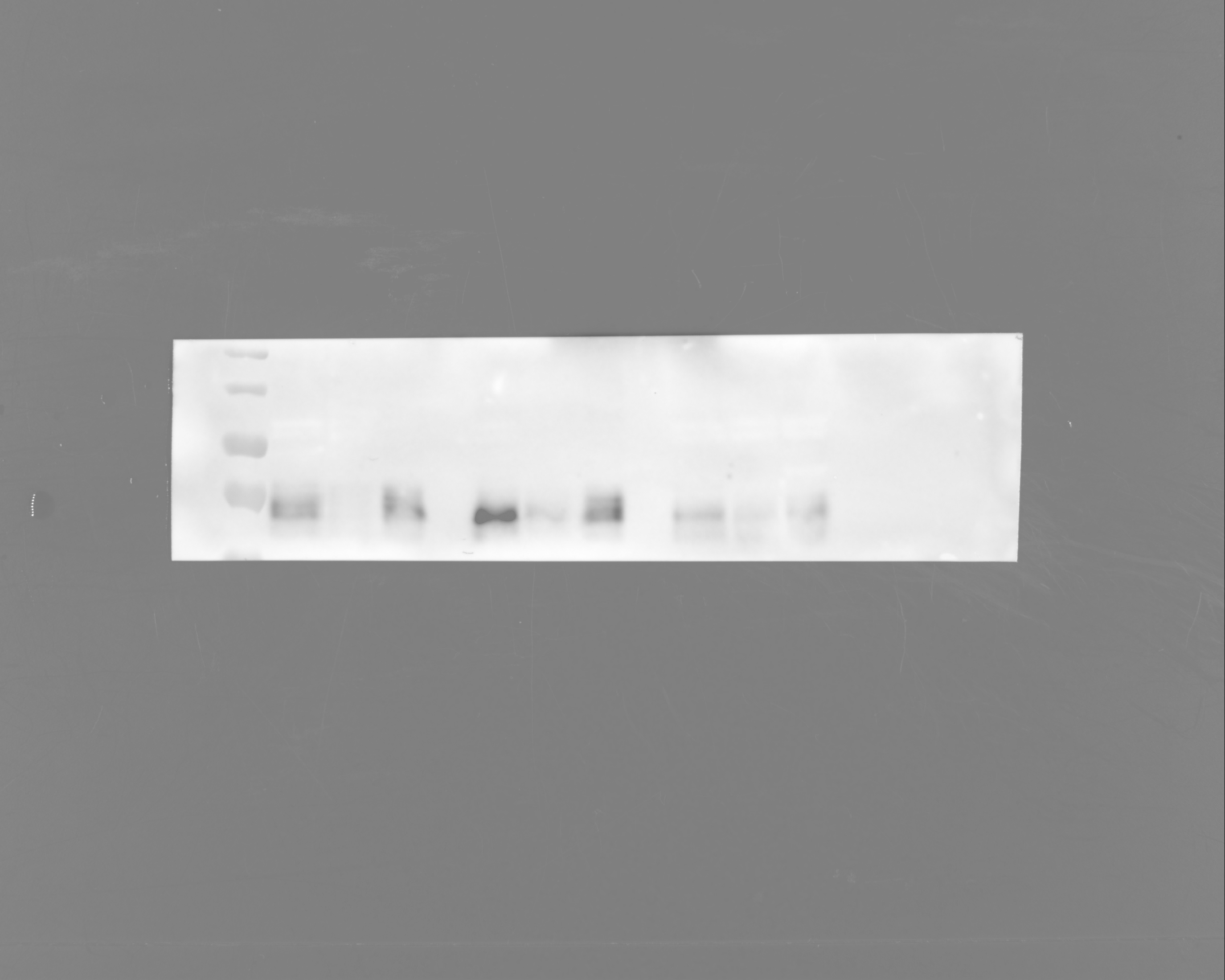

Supplement: Figure 5—source data 6. [file elife-78874-fig5-data6.zip › Figure 5-source data 6/Figure 5-source data 6.tif]

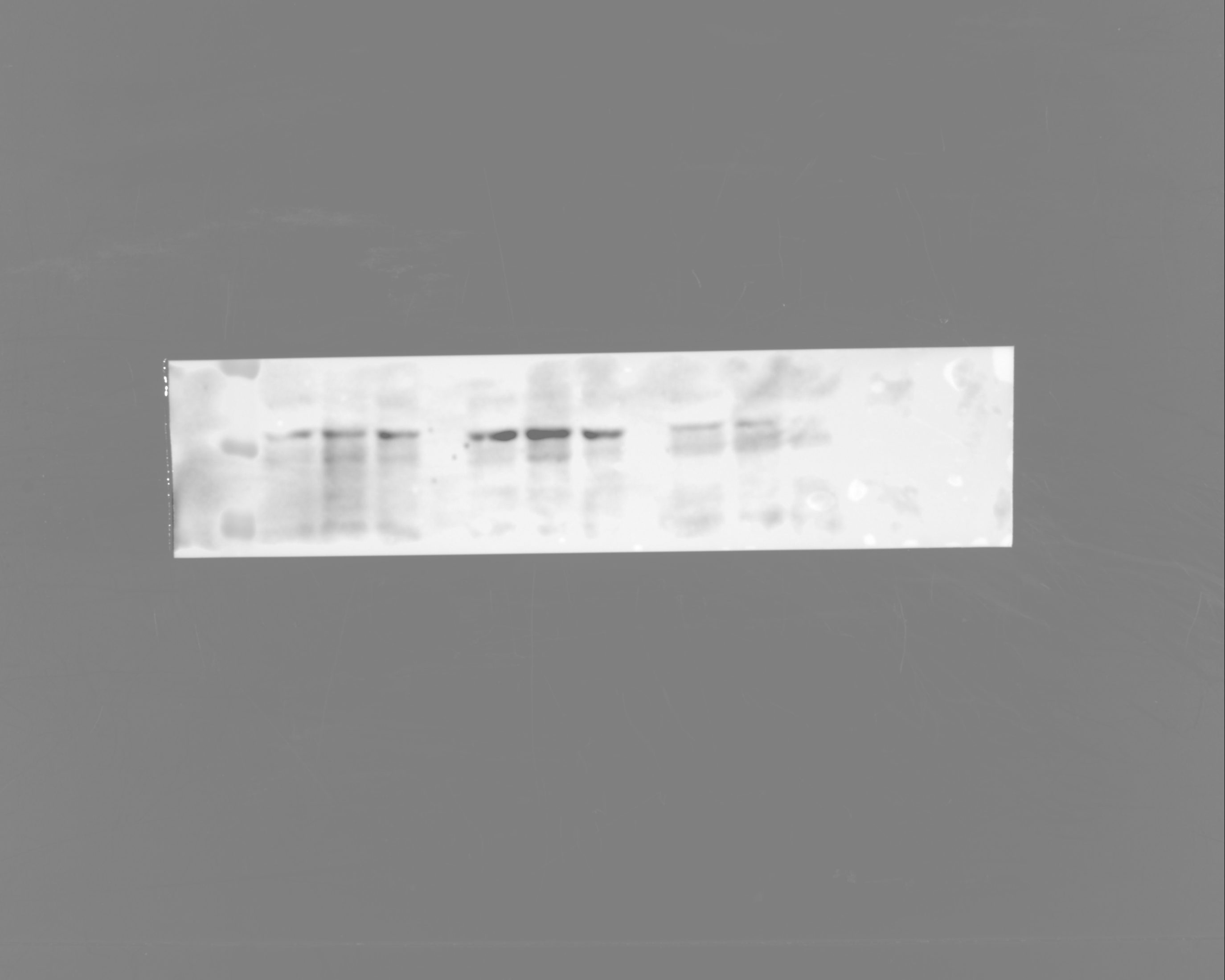

Supplement: Figure 5—source data 7. [file elife-78874-fig5-data7.zip › Figure 5-source data 7/Figure 5-source data 7.tif]

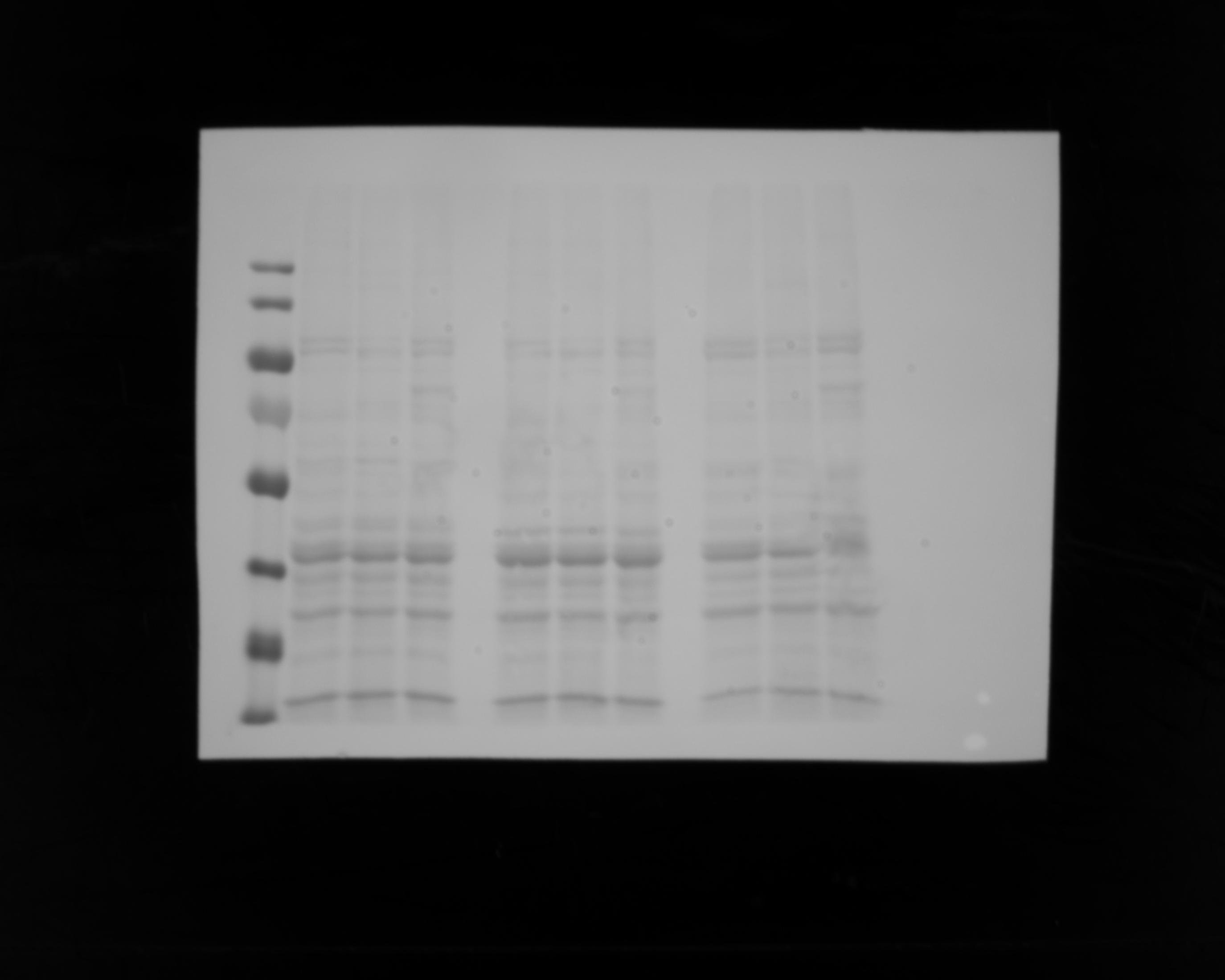

Supplement: Figure 5—source data 8. [file elife-78874-fig5-data8.zip › Figure 5-source data 8/Figure 5-source data 8.tif]

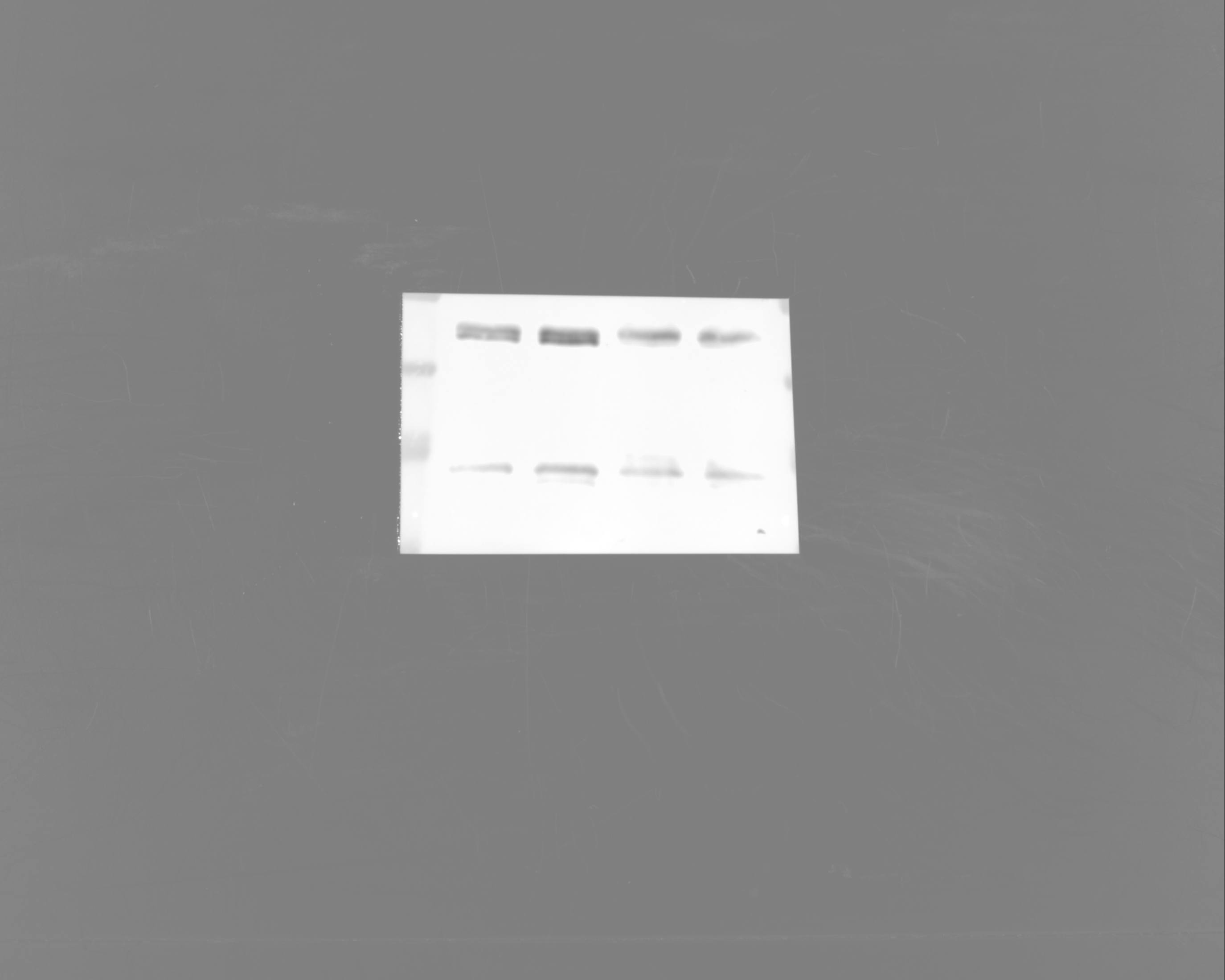

Supplement: Figure 8—source data 1. [file elife-78874-fig8-data1.zip › Figure 8-source data 1/Figure 8-source data 1.tif]

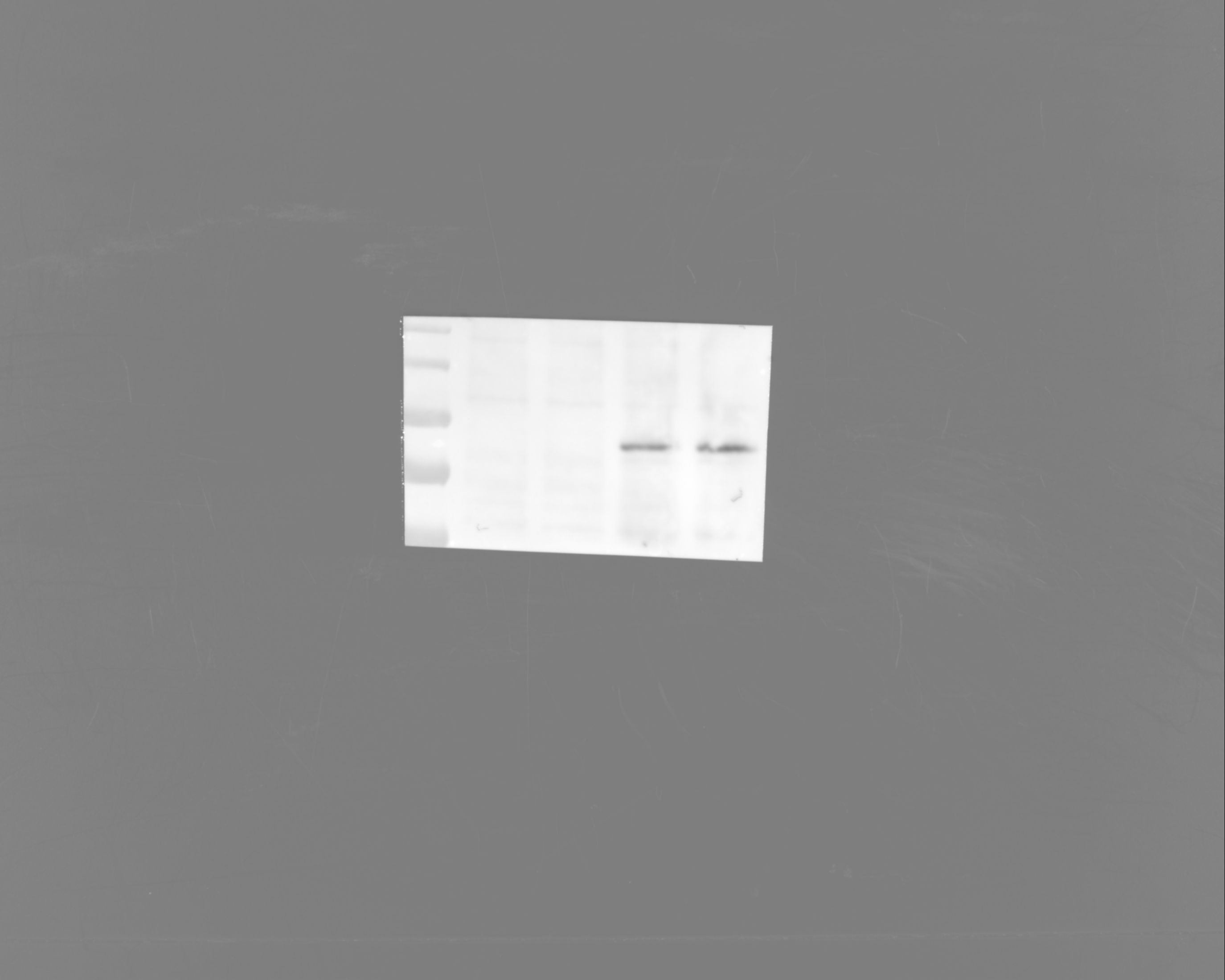

Supplement: Figure 8—source data 2. [file elife-78874-fig8-data2.zip › Figure 8-source data 2/Figure 8-source data 2.tif]

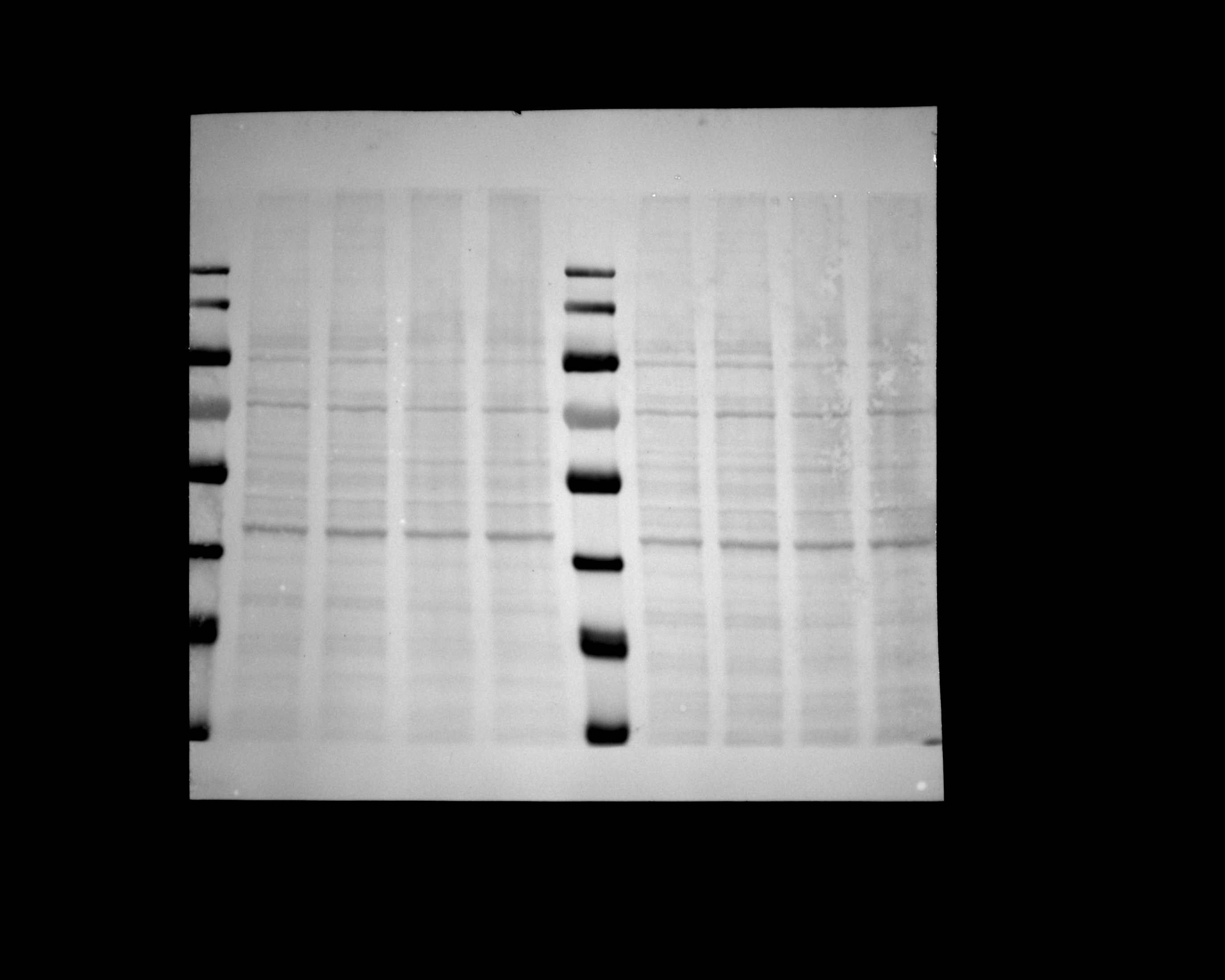

Supplement: Figure 8—source data 3. — GSK3B blot is derived from the left part and JPh from right part of the membrane. [file elife-78874-fig8-data3.zip › Figure 8-source data 3/Figure 8-source data 3.tif]
