# Supplementary material for: Muscle calcium stress cleaves junctophilin1, unleashing a gene regulatory program predicted to correct glucose dysregulation
Source: eLife. 2023 Feb 1;12:e78874. doi: 10.7554/eLife.78874 (PMC9891728; doi:10.7554/eLife.78874)

Figure 1-figure supplement 1-source data 1: following raw blot of JPh1 (abA) is used for Supplemental fig1 to fig. 1.

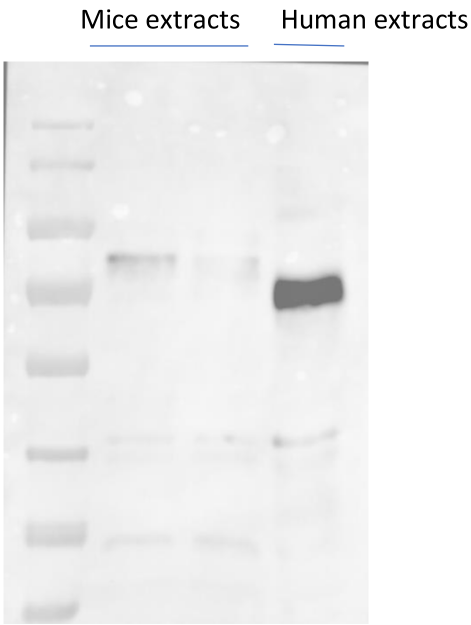

Figure 1-figure supplement 1-source data 1: Above blot is derived from right part of following ponceau stain membrane.

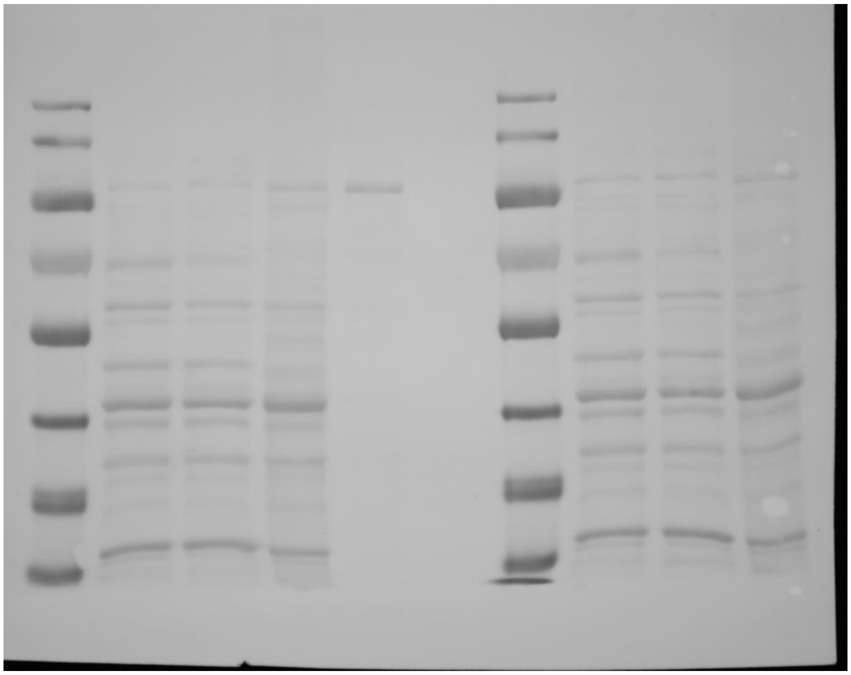

Supplement: Figure 1—figure supplement 1—source data 1. [file elife-78874-fig1-figsupp1-data1.zip › Figure 1-figure supplement 1-source data 1/Annoted Figure 1 supplement 1 source data.pdf]
