# Supplementary material for: Muscle calcium stress cleaves junctophilin1, unleashing a gene regulatory program predicted to correct glucose dysregulation
Source: eLife. 2023 Feb 1;12:e78874. doi: 10.7554/eLife.78874 (PMC9891728; doi:10.7554/eLife.78874)

Figure 1-figure supplement 2-source data 1: Different amounts of human muscle total are blotted with Junctophilin abA as presented in supplement figure 2 for Fig. 1

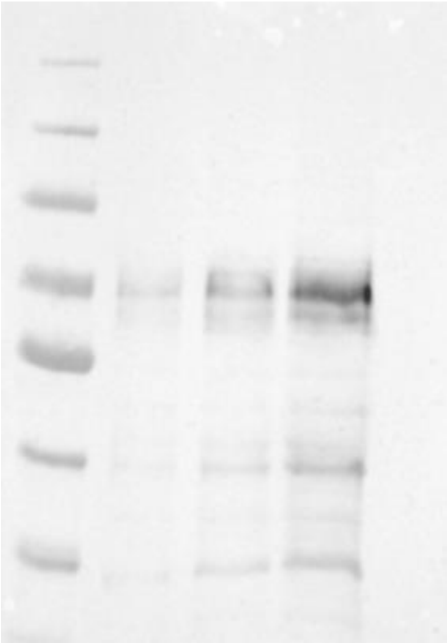

Figure 1-figure supplement 2-source data 2: Different amounts of human muscle total are blotted with Junctophilin abB as presented in supplement figure 2 for Fig. 1

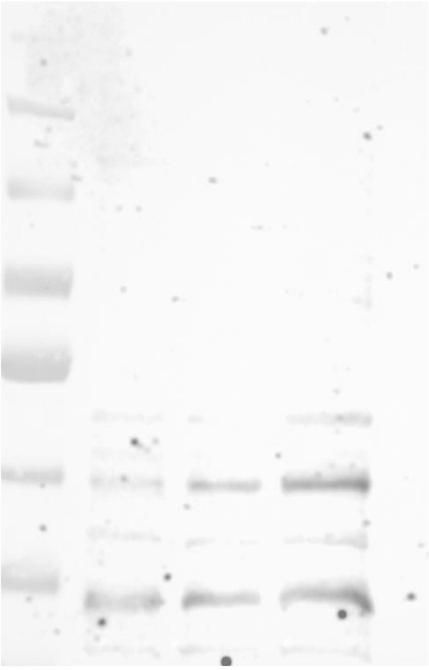

Figure 1-figure supplement 2-source data 2: Above blots are derived from following ponceau stain normalizing blot. Junctophilin abA blot is obtained from the left part of following ponceau stain membrane and Junctophilin abB blot is obtained from right part of the membrane.

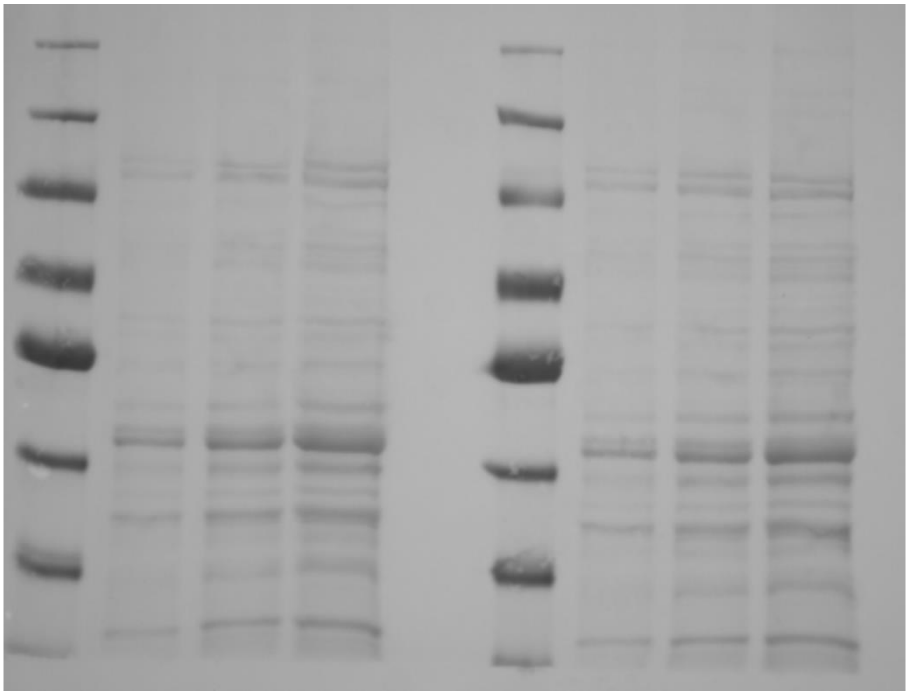

Supplement: Figure 1—figure supplement 2—source data 1. [file elife-78874-fig1-figsupp2-data1.zip › Figure 1-figure supplement 2-source data 1/Annoted Figure 1-figure supplement 2 source data.pdf]
