# Supplementary material for: Muscle calcium stress cleaves junctophilin1, unleashing a gene regulatory program predicted to correct glucose dysregulation
Source: eLife. 2023 Feb 1;12:e78874. doi: 10.7554/eLife.78874 (PMC9891728; doi:10.7554/eLife.78874)

Figure 3-source data 1

JPh1 raw blot shown in figure 3D

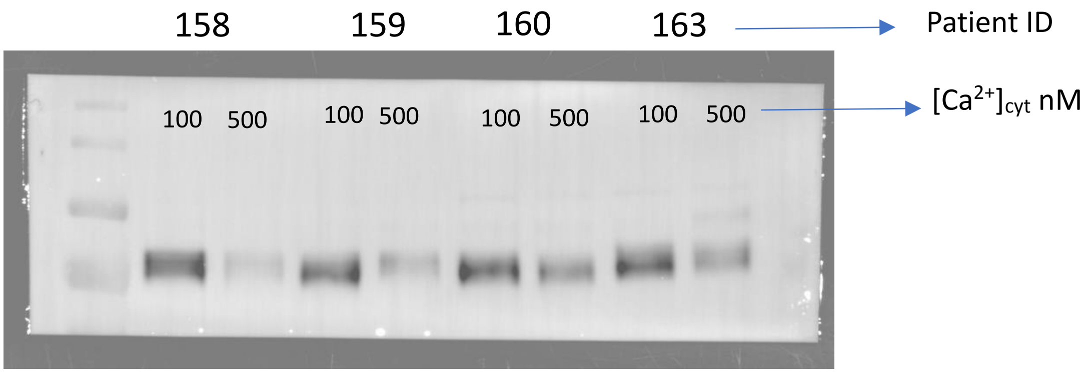

Figure 3-source data 2

Normalizing ponceau stain whole blot for JPh1 shown in figure 3D

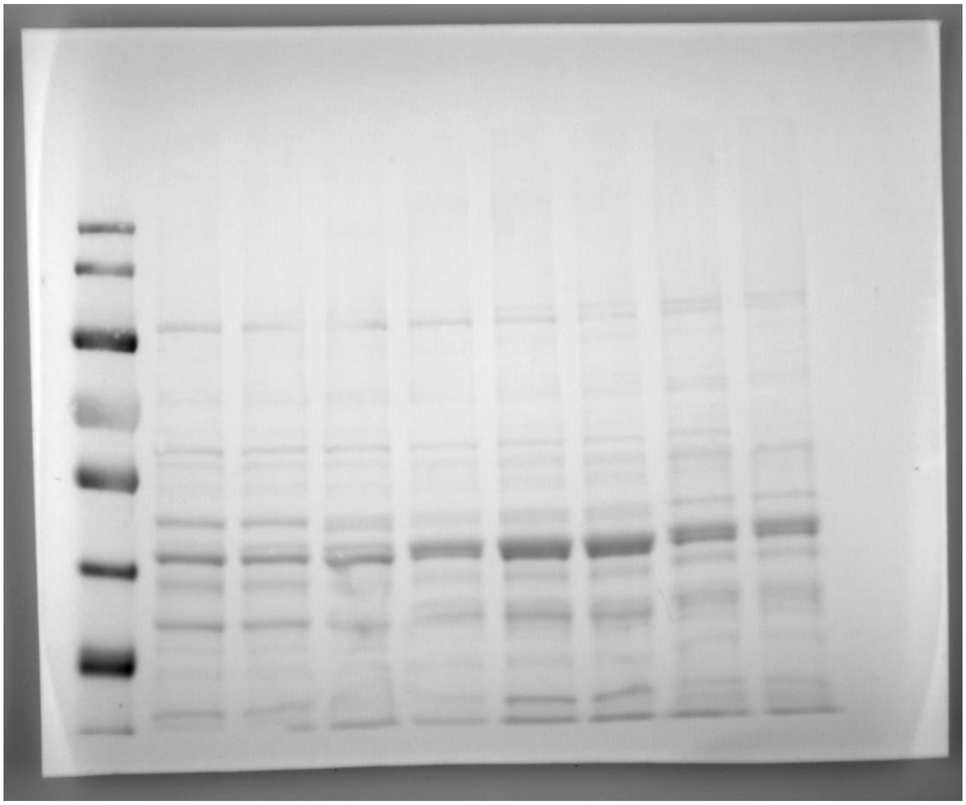

Figure 3-source data 3

Boxed region of following raw blot is shown as JPh44 detected by JPh1 abB in figure 3E. The bands of upper molecular weight (particularly at 70 kDa) are resulted from earlier incubation of same membrane with JPh1 abA and its improper stripping. Last 3 lanes in the blot are unrelated to the experiment.

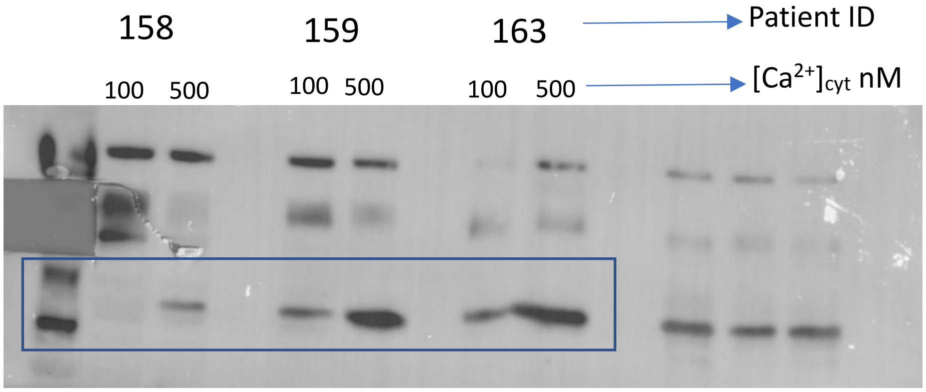

Figure 3-source data 4

Normalizing ponceau stain whole blot for JPh44 shown in figure 3E

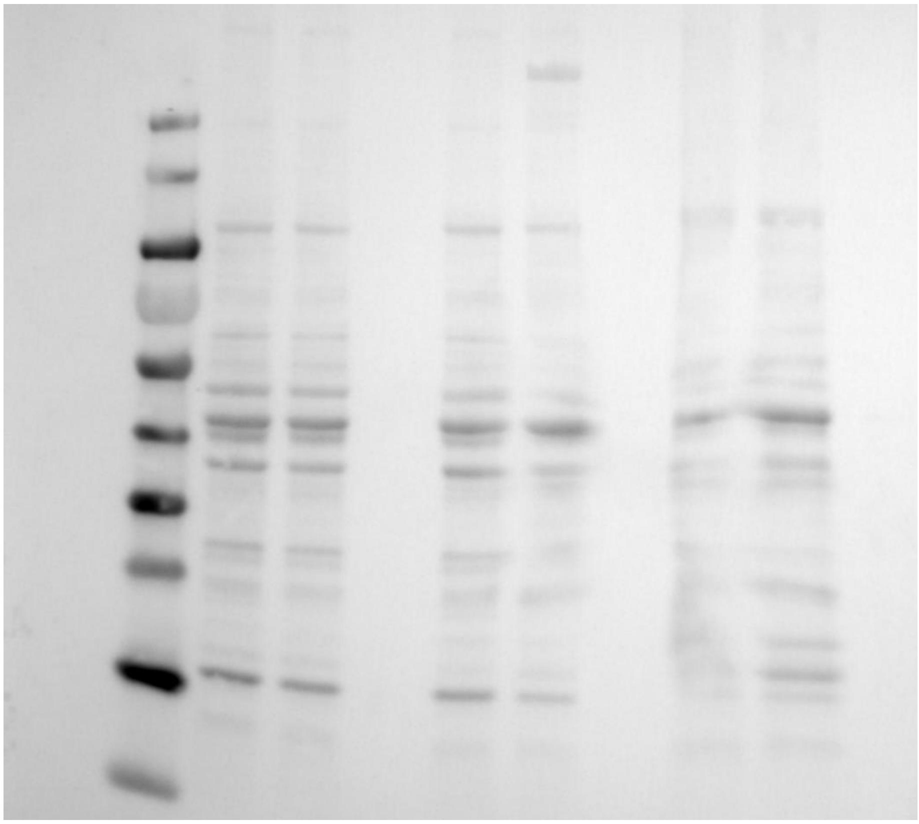

Figure 3-source data 5

MHN and MHS nuclear fractions JPh44 raw blot shown in figure 3G

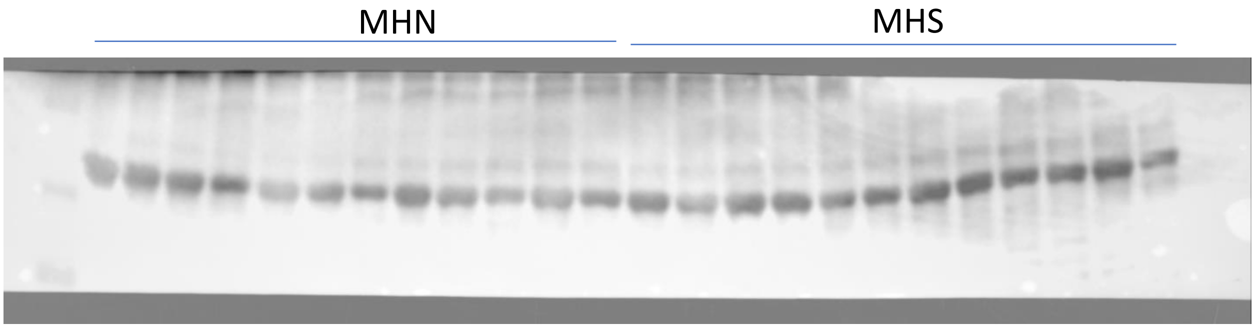

Figure 3-source data 6

Normalizing ponceau stain whole blot for JPh44 of nuclear fractions shown in figure 3G

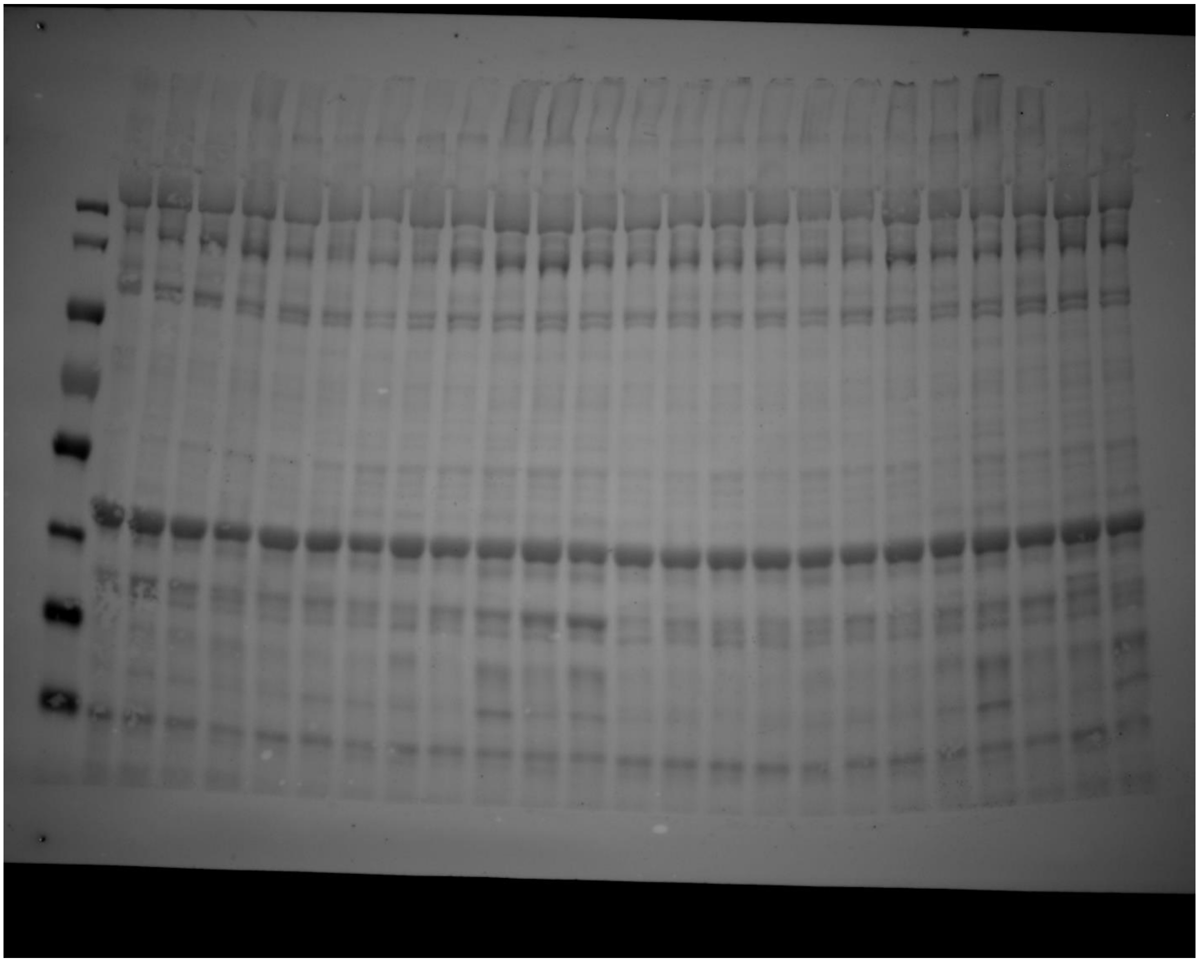

Supplement: Figure 3—source data 1. [file elife-78874-fig3-data1.zip › Figure 3-source data 1/Annoted Figure 3-source data.pdf]
