# Supplementary material for: Muscle calcium stress cleaves junctophilin1, unleashing a gene regulatory program predicted to correct glucose dysregulation
Source: eLife. 2023 Feb 1;12:e78874. doi: 10.7554/eLife.78874 (PMC9891728; doi:10.7554/eLife.78874)

Figure 4

Figure 4- source data 1: Boxed region in below is shown as calpain blot for figure 4D. Lower part of the blot indicating autolyzed calpain fragments of small molecular weight is shown in supplement figure 2 to Figure 4.

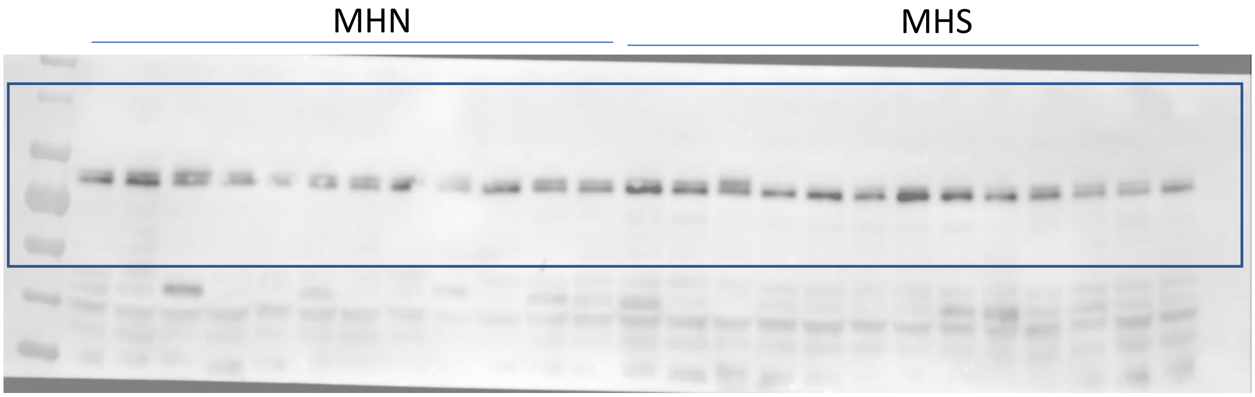

Figure 4- source data 2: Normalizing ponceau stain blot l for Calpain blot shown in figure 4D

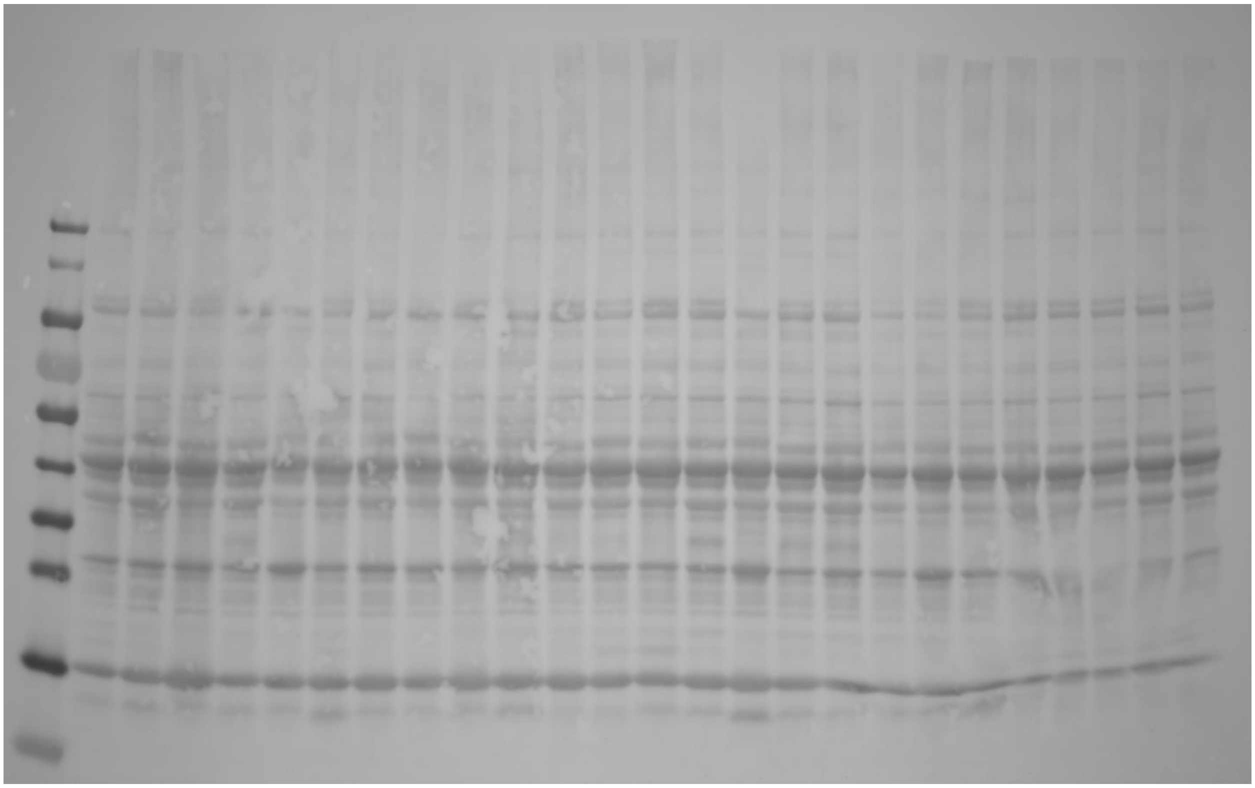

Supplement: Figure 4—source data 1. — Lower part of the blot, with calpain fragments of small molecular weight is shown in Figure 4—figure supplement 2. [file elife-78874-fig4-data1.zip › Figure 4- source data 1/Annoted figure 4- source data.pdf]
