# Supplementary material for: Muscle calcium stress cleaves junctophilin1, unleashing a gene regulatory program predicted to correct glucose dysregulation
Source: eLife. 2023 Feb 1;12:e78874. doi: 10.7554/eLife.78874 (PMC9891728; doi:10.7554/eLife.78874)

Figure 4-figure supplement 2 source data1 : Boxed region in below blot is indicated as autolyzed calpain fragments of small molecular weight.

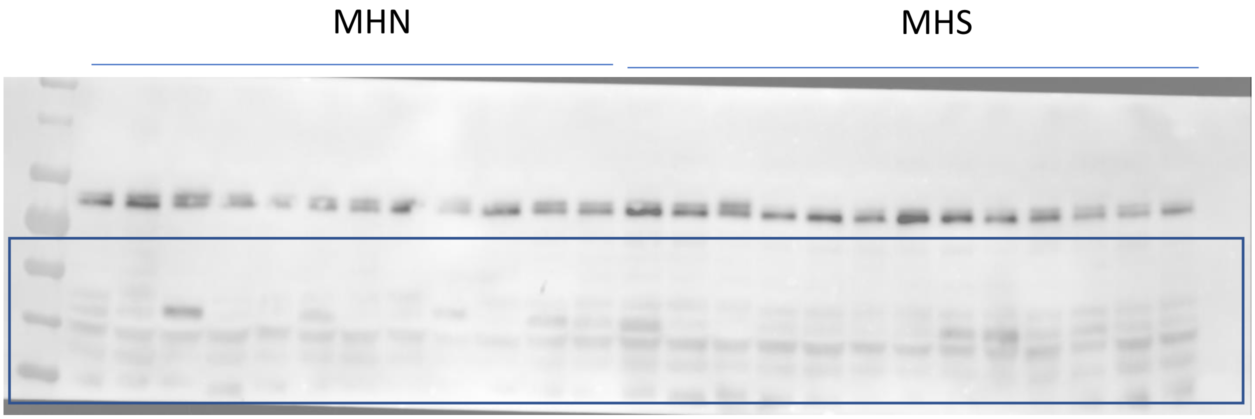

Figure 4- figure supplement 2 source data 2: Normalizing ponceau stain blot for the above boxed Calpain blot presented as Figure 4- figure supplement 2 in the manuscript.

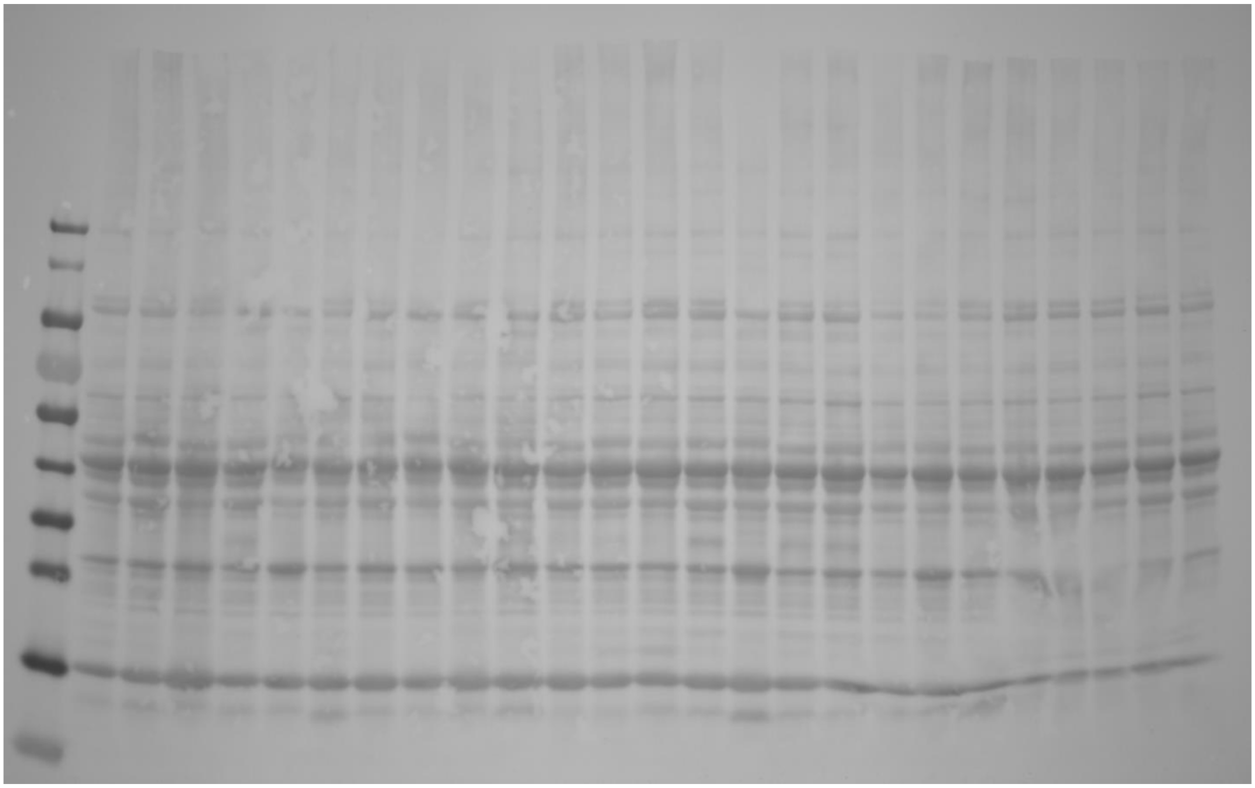

Supplement: Figure 4—figure supplement 2—source data 1. [file elife-78874-fig4-figsupp2-data1.zip › Figure 4-figure supplement 2-source data 1/Annoted Figure 4-figure supplement 2 source data.pdf]
