# Supplementary material for: Muscle calcium stress cleaves junctophilin1, unleashing a gene regulatory program predicted to correct glucose dysregulation
Source: eLife. 2023 Feb 1;12:e78874. doi: 10.7554/eLife.78874 (PMC9891728; doi:10.7554/eLife.78874)

Figure 5

Figure 5-source data 1: JPh raw blot incubated with JPh abA shown in figure 5A

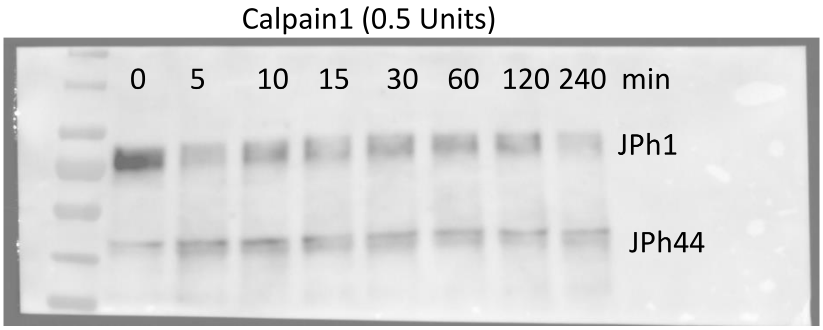

Figure 5-source data 2: Normalizing ponceau stain blot for JPh blot shown in figure 5A

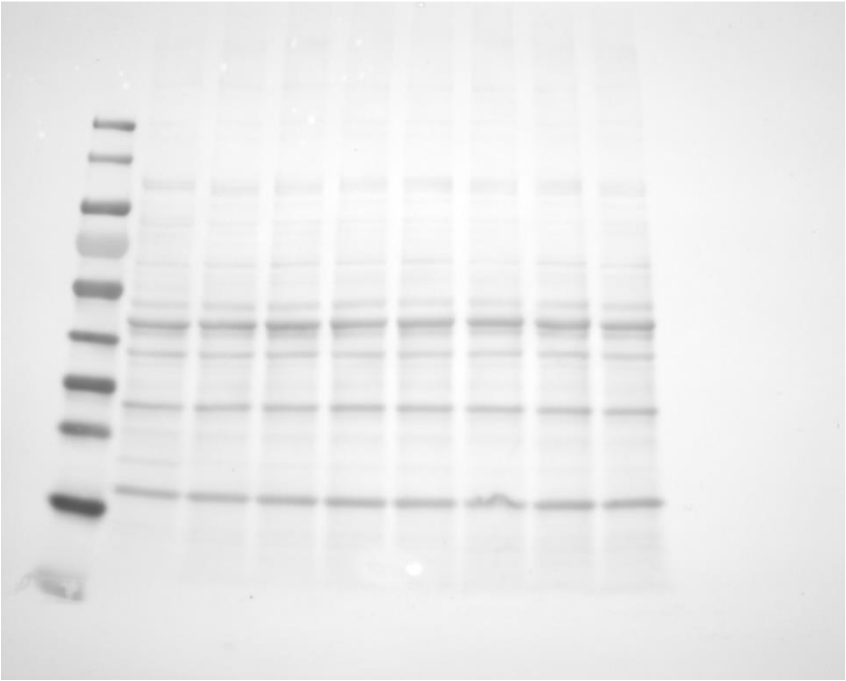

Figure 5-source data 3: Boxed area of following JPh1 blot is shown in figure 5C

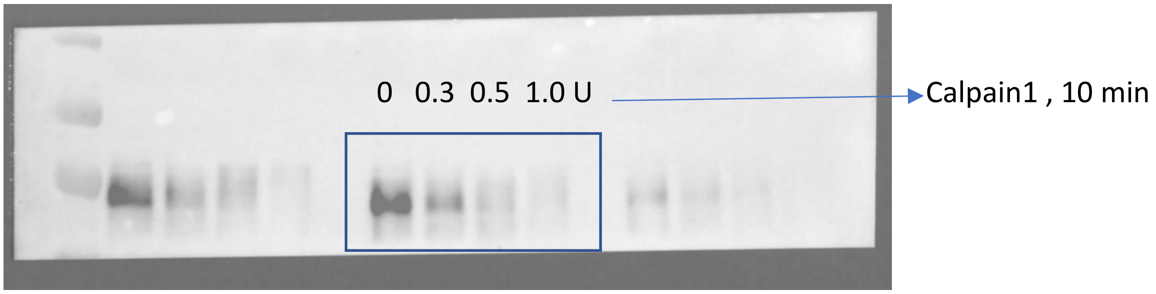

Figure 5-source data 4: Boxed area of following JPh44 blot is shown in figure 5C

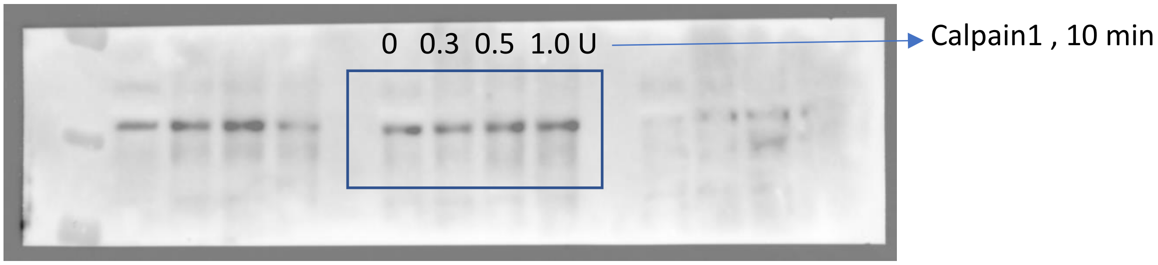

Figure 5-source data 5: Following normalizing ponceau stain blot is used for above figure shown in 5C

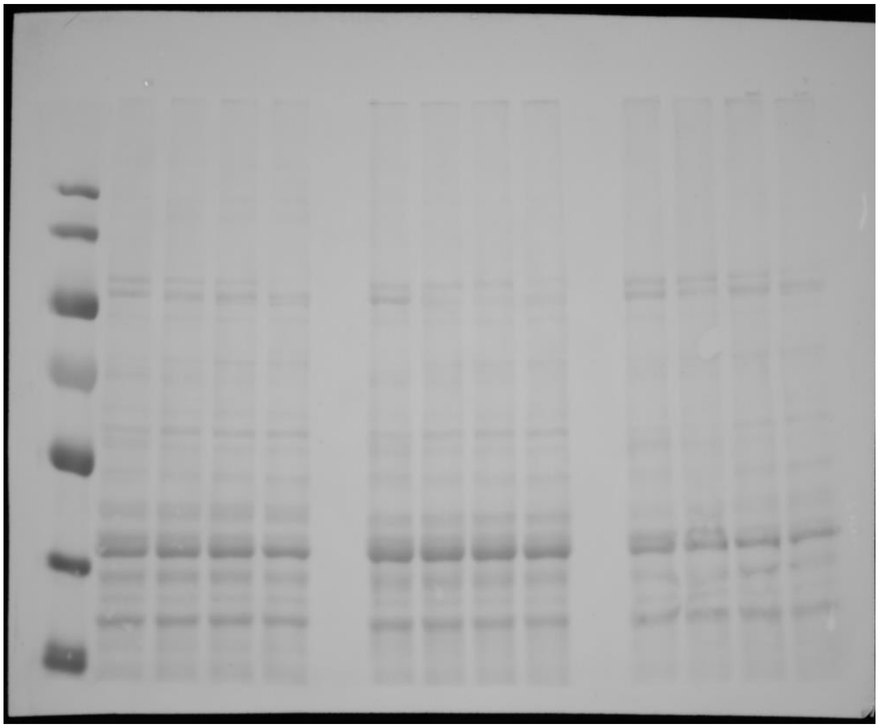

Figure 5-source data 6: Boxed area of following JPh1 blot is shown in figure 5E

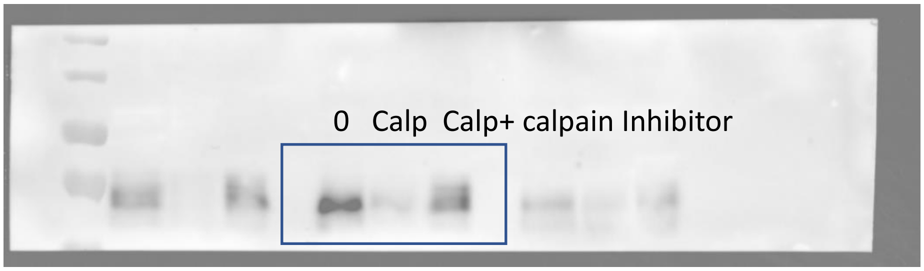

Figure 5-source data 7: Boxed area of following JPh44 blot is shown in figure 5E

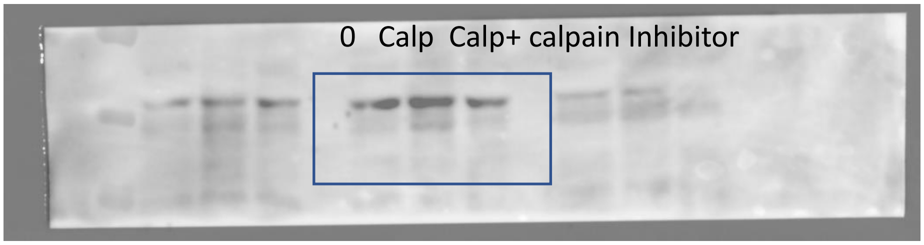

Following normalizing ponceau stain blot is used for above figure shown in 5E

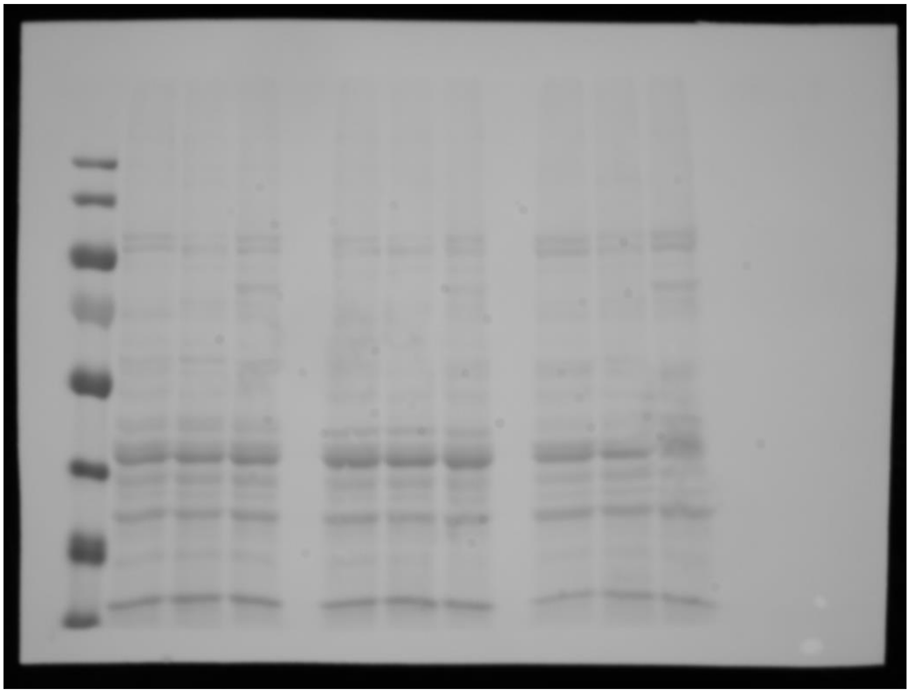

Supplement: Figure 5—source data 1. [file elife-78874-fig5-data1.zip › Figure 5-source data 1/Annoted figure 5-source data.pdf]
