# Supplementary material for: Muscle calcium stress cleaves junctophilin1, unleashing a gene regulatory program predicted to correct glucose dysregulation
Source: eLife. 2023 Feb 1;12:e78874. doi: 10.7554/eLife.78874 (PMC9891728; doi:10.7554/eLife.78874)

Figure 8-source data 1: following boxed region is shown as GSK3b levels in cell extracts transfected with control (empty vector) plasmid and GFP-Δ(1-240) JPh1 plasmid and detected with JPh1 antibody (abA) as shown figure 8E.

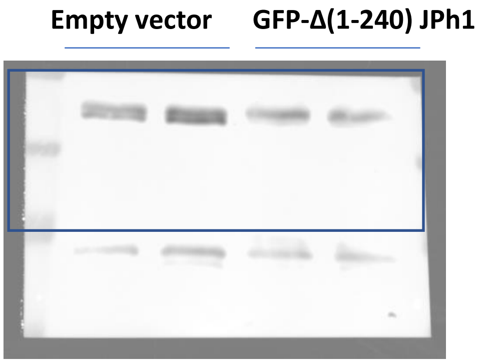

Figure 8-source data 2: GFP-Δ(1-240) JPh1 levels are detected in cell extracts transfected with control (empty vector) plasmid and GFP-Δ(1-240) JPh1 plasmid with JPh1 antibody (abA) as shown figure 8E.

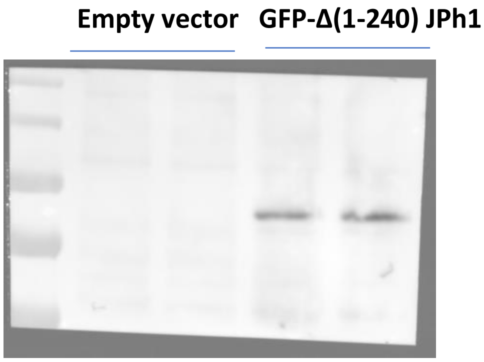

Figure 8-source data 3: Above blots are derived from following ponceau stain normalizing blot. GSK3B blot is obtained from the left part of following ponceau stain membrane and JPH1 blot is obtained from right part of the membrane.

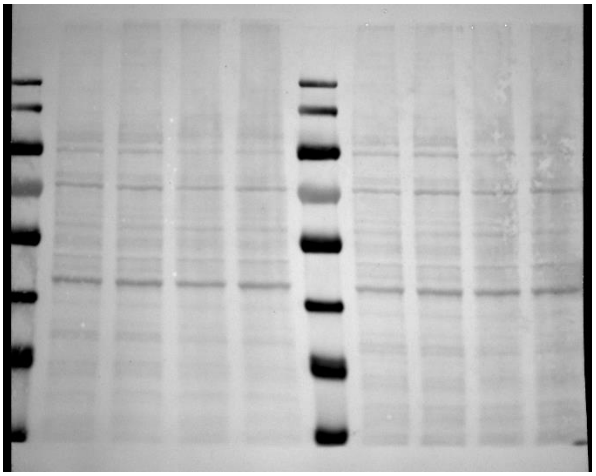

Supplement: Figure 8—source data 1. [file elife-78874-fig8-data1.zip › Figure 8-source data 1/Annoted Figure 8- source data.pdf]
