# Supplementary material for: Muscle calcium stress cleaves junctophilin1, unleashing a gene regulatory program predicted to correct glucose dysregulation
Source: eLife. 2023 Feb 1;12:e78874. doi: 10.7554/eLife.78874 (PMC9891728; doi:10.7554/eLife.78874)
Supplement: Supplementary file 1. [file elife-78874-supp1.docx]

## Supplementary File 1. Supplemental Table

| **Patient ID** | **Age/Gender** | **Ethnicity** | **CHCT status** |
| --- | --- | --- | --- |
| 81 | 33/F | First nation/indigenous | HN |
| 96 | 41/M | Caucasian | HN |
| 98 | 39/M | Caucasian | HN |
| 99 | 36/M | Caucasian | HN |
| 102 | 43/M | Caucasian | HN |
| 103 | 35/ F | Caucasian | HN |
| 105 | 29/M | Caucasian | HN |
| 108 | 20/M | Caucasian | HH |
| 110 | 21/F | Caucasian | HN |
| 115 | 36/M | Caucasian | HH |
| 117 | 39/F | Caucasian | HN |
| 118 | 31/M | East Asian | HN |
| 120 | 39/M | Caucasian | HH |
| 121 | 32/M | Caucasian | HH |
| 122 | 38/M | Caucasian | HN |
| 124 | 30/M | Caucasian | HH |
| 128 | 43/F | Caucasian | HN |
| 137 | 39/F | Hispanic | HH |
| 140 | 62/F | Ashkenazie | HN |
| 142 | 39/F | Caucasian | HH |
| 143 | 38/F | Caucasian | HH |
| 144 | 48/M | Caucasian | HN |
| 145 | 24/M | Indian | HH |
| 147 | 50/M | Caucasian | HH |
| 148 | 47/M | Caucasian | HN |
| 149 | 55/M | Indigenous | HN |
| 152 | 34/M | Caucasian | HN |
| 154 | 33/M | Ashkenazie | HH |
| 155 | 34/F | Caucasian | HN |
| 156 | M | Hispanic | HS |
| 158 | 20/M | Caucasian | HN |
| 159 | 18/M | Caucasian | HH |
| 160 | 18/M | Caucasian | HS |
| 161 | 39/M | Caucasian | HN |
| 162 | 25/F | Caucasian | HH |
| 163 | 38/M | Caucasian | HN |
| 164 | 31/F | First nation/indigenous | HN |
| 165 | 35/M | Caucasian | HN |
| 166 | 21/M | Caucasian | HS |
| 167 | 39/M | Caucasian | HH |
| 168 | 21/M | Caucasian | HH |
| 169 | 48/M | Presumed Caucasian  ( Adopted) | HH |
| 170 | 24/F | Caucasian | HN |
| 172 | 34/F | Caucasian | HN |
| 173 | 38/M | Caucasian | HH |
| 175 | 29/F | Caucasian | HH |
| 176 | 30/F | Caucasian | HN |
| 179 | 22/F | Chinese | HS |
| 180 | 21/F | Caucasian | HN |
| 184 | 60/F | Caucasian | HN |
| 187 | 35/M | Caucasian | HN |
| 189 | 59/M | Caucasian | HS |
| 190 | 54/M | Caucasian | HH |
| 194 | 24/F | Caucasian | HN |
| 223 | 30/F | Caucasian | HN |
| 224 | 48/M | Caucasian | HS |

Supplemental Table. Demographic information for all patients whose samples were analyzed for this study. Col 1: ID #; Col 2: Test date; Col 3: Age/sex; Col 4: ethnicity or race; Col 5: CHCT diagnostic category, nomenclature introduced by (22): HN, normal; HH, hyperreactive to halothane only; HS, hyperreactive to both halothane and caffeine. * First nation/indigenous.
